# Supplementary material for: Future nitrogen availability and its effect on carbon sequestration in Northern Eurasia
Source: Nat Commun. 2019 Jul 9;10:3024. doi: 10.1038/s41467-019-10944-0 (PMC6616380; doi:10.1038/s41467-019-10944-0)
Supplement: Supplementary file 1 — Supplementary Information [file 41467_2019_10944_MOESM1_ESM.pdf]

Supplementary Information

Future nitrogen availability and its effect on carbon sequestration in Northern Eurasia

Kicklighter *et al.*

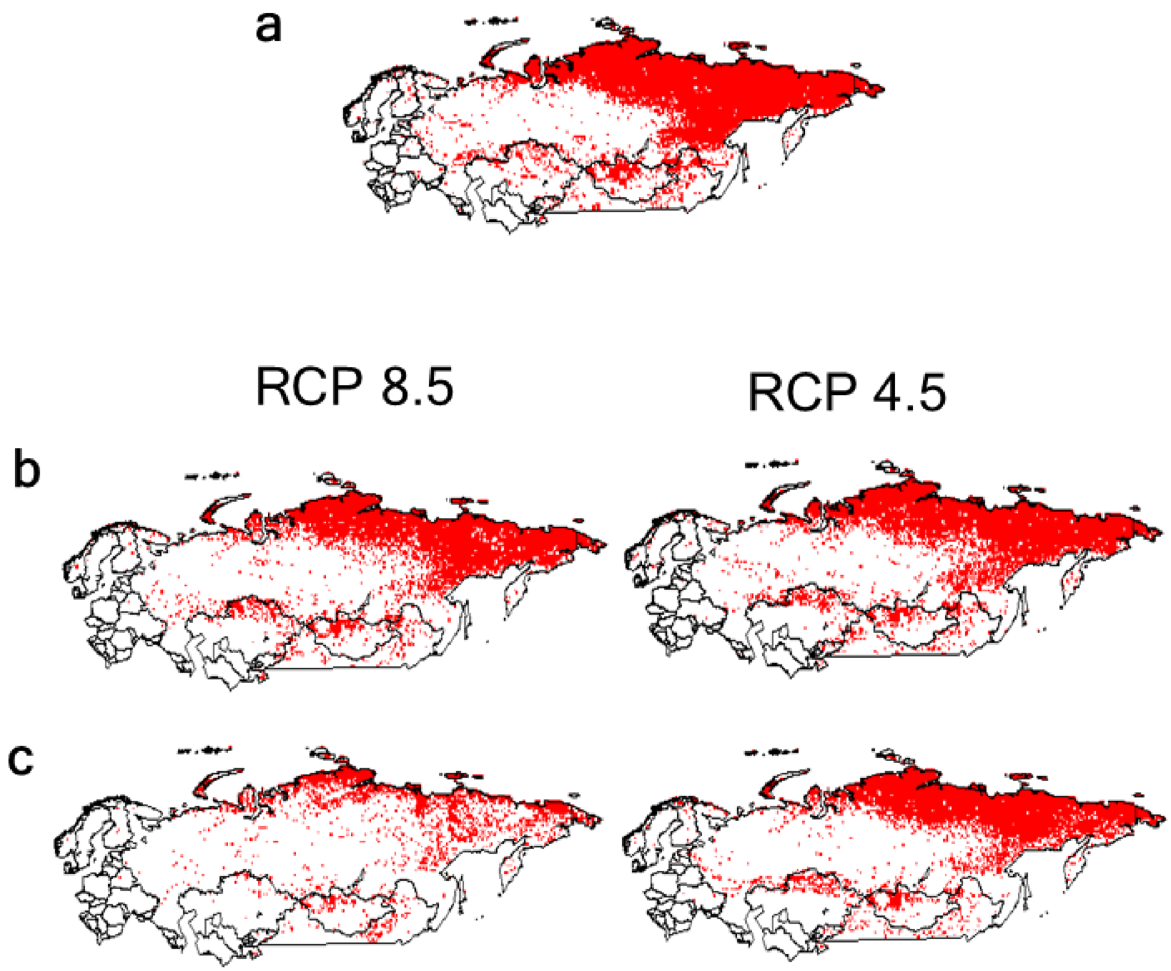

Supplementary Figure 1. Projected changes in the distribution of permafrost during the 21st century. **a** Permafrost distribution during the year 2000. **b** Permafrost distribution during the year 2050 using the RCP8.5 and RCP4.5 global change scenarios. **c** Same as (**b**), for year 2100. Permafrost is assumed to occur (red) where the maximum monthly soil temperature at 2 m depth is never above 0° C during the specified year.

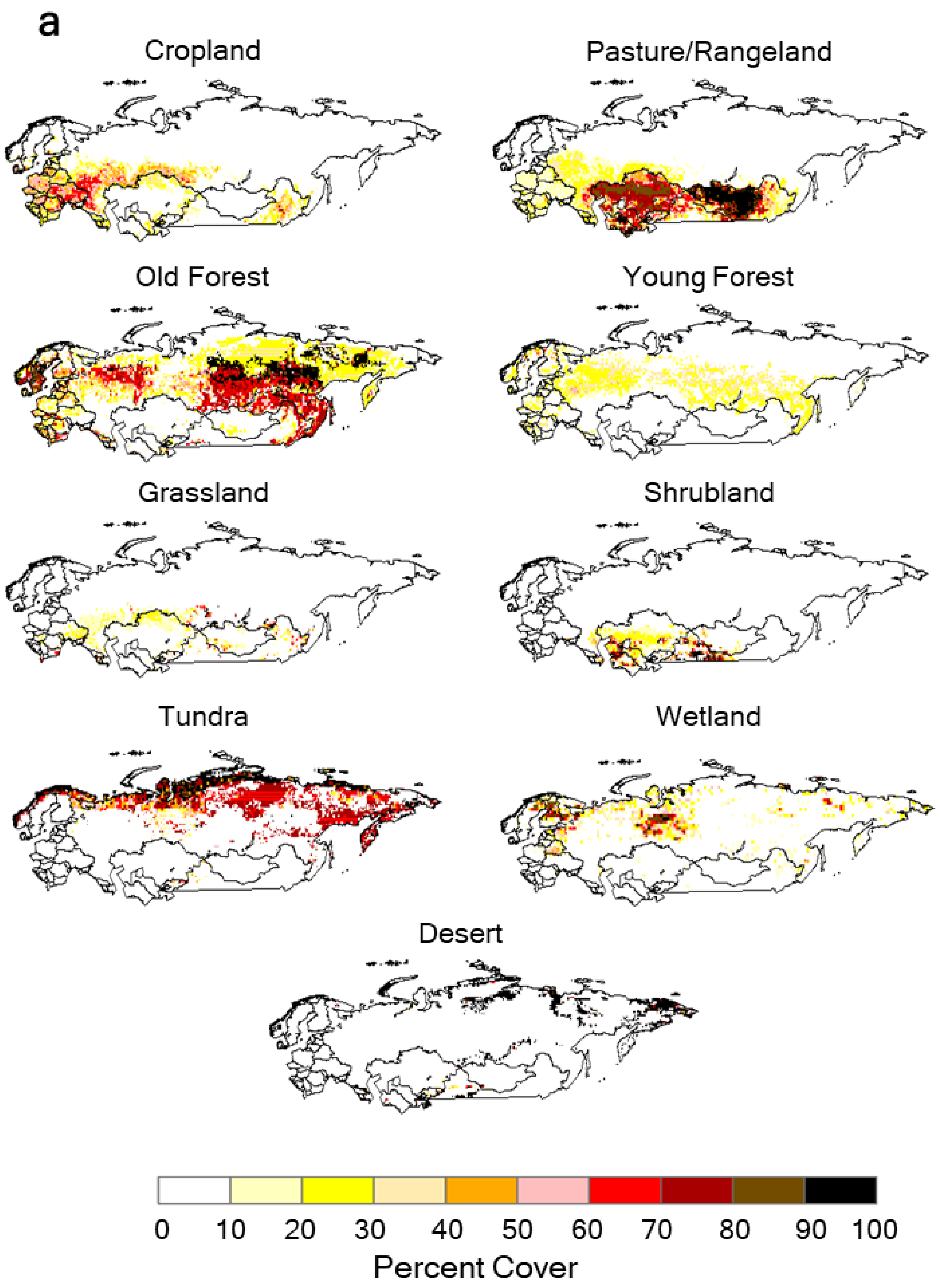

Supplementary Figure 2. Projected changes in land cover across Northern Eurasia over the 21st century. **a** Land cover distribution during year 2000. **b** Changes in land cover from year 2000 as projected by the RCP8.5 scenario for the year 2100. **c** Same as (**b**), but for the RCP4.5 scenario.

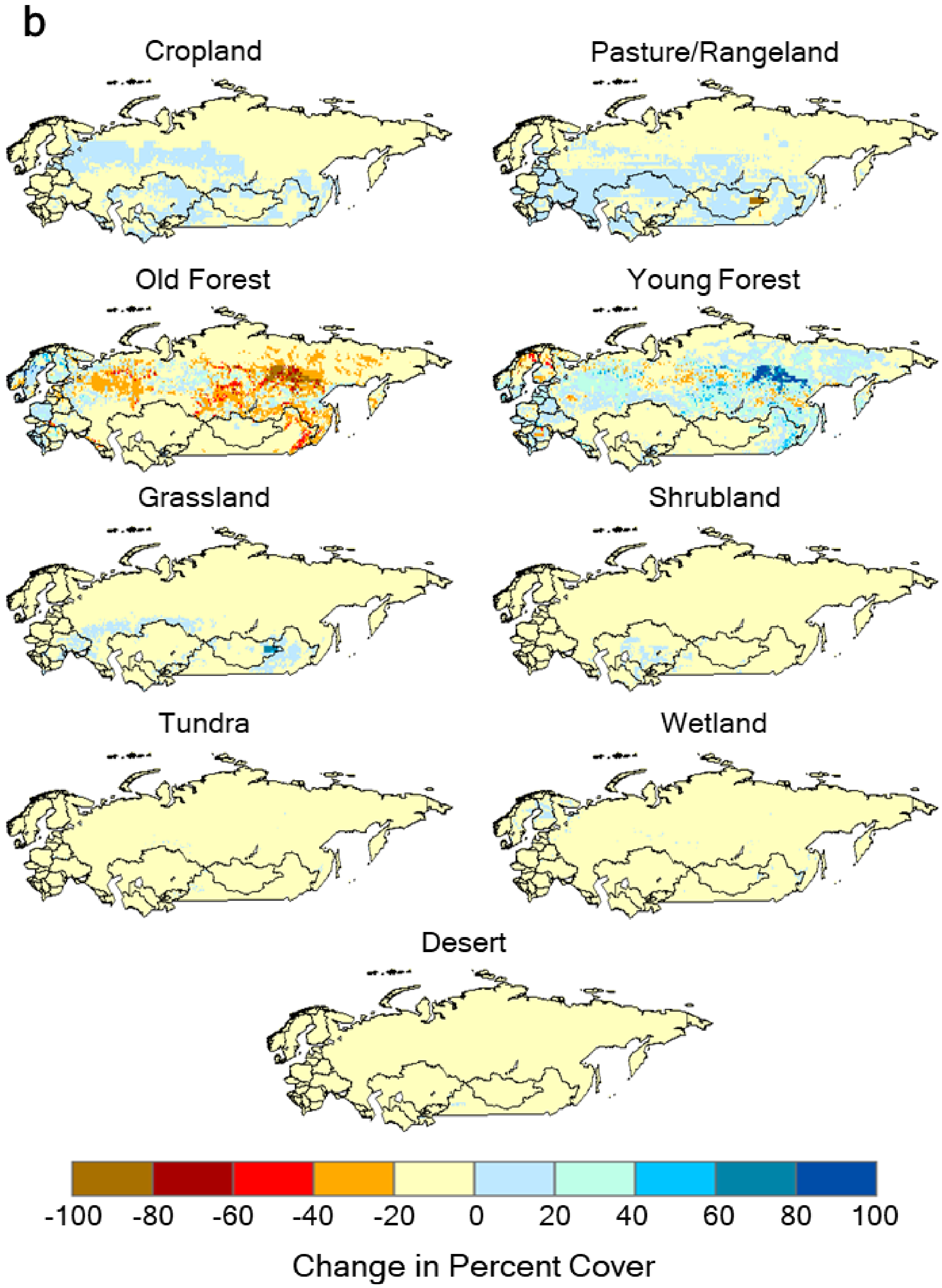

16 **Supplementary Figure 2 (continued).** Projected changes in land cover across Northern Eurasia
17 over the 21st century. **a** Land cover distribution during year 2000. **b** Changes in land cover from
18 year 2000 as projected by the RCP8.5 scenario for the year 2100. **c** Same as (**b**), but for the
19 RCP4.5 scenario.

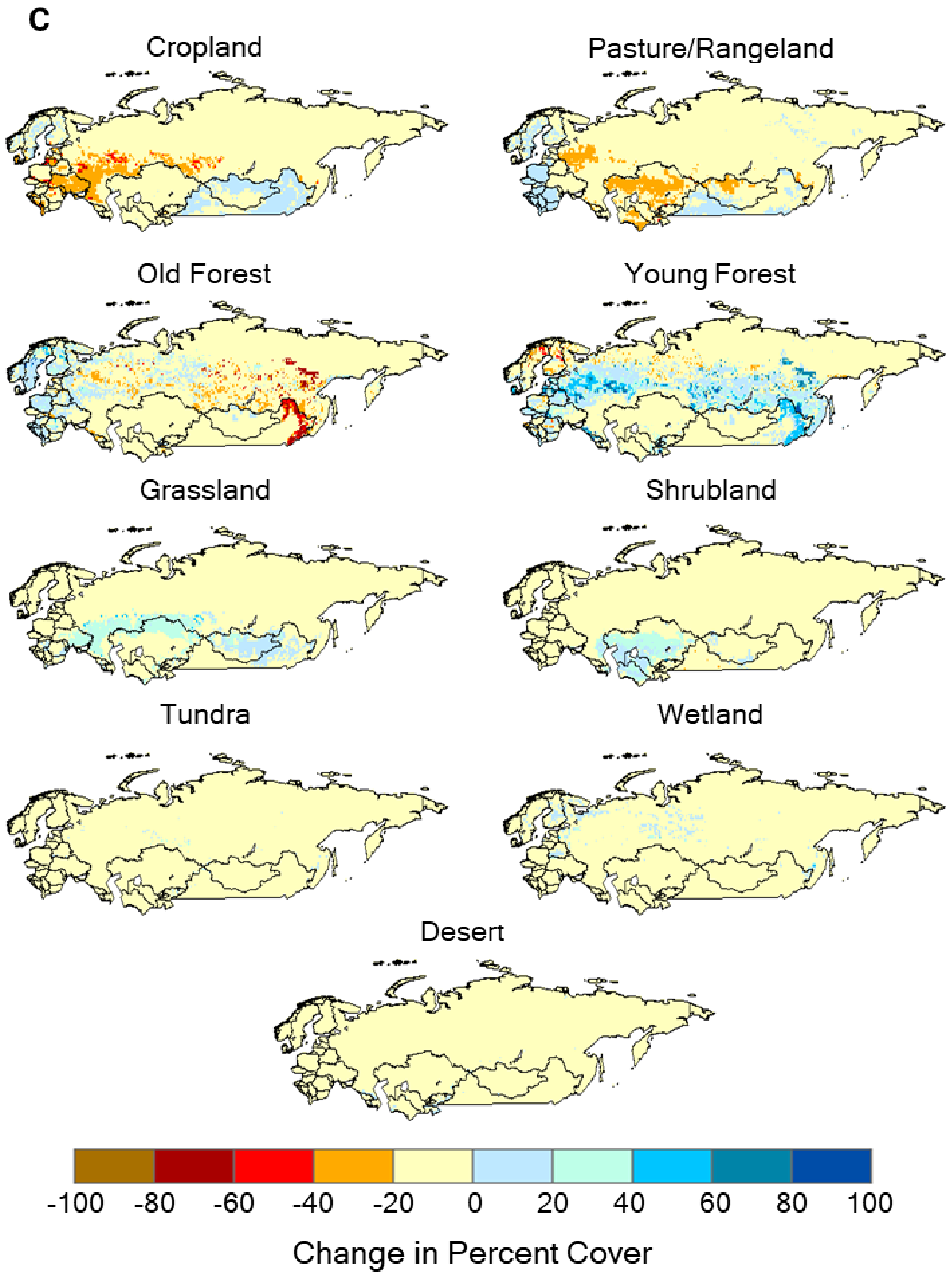

Supplementary Figure 2 (continued). Projected changes in land cover across Northern Eurasia over the 21st century. **a** Land cover distribution during year 2000. **b** Changes in land cover from year 2000 as projected by the RCP8.5 scenario for the year 2100. **c** Same as (**b**), but for the RCP4.5 scenario.

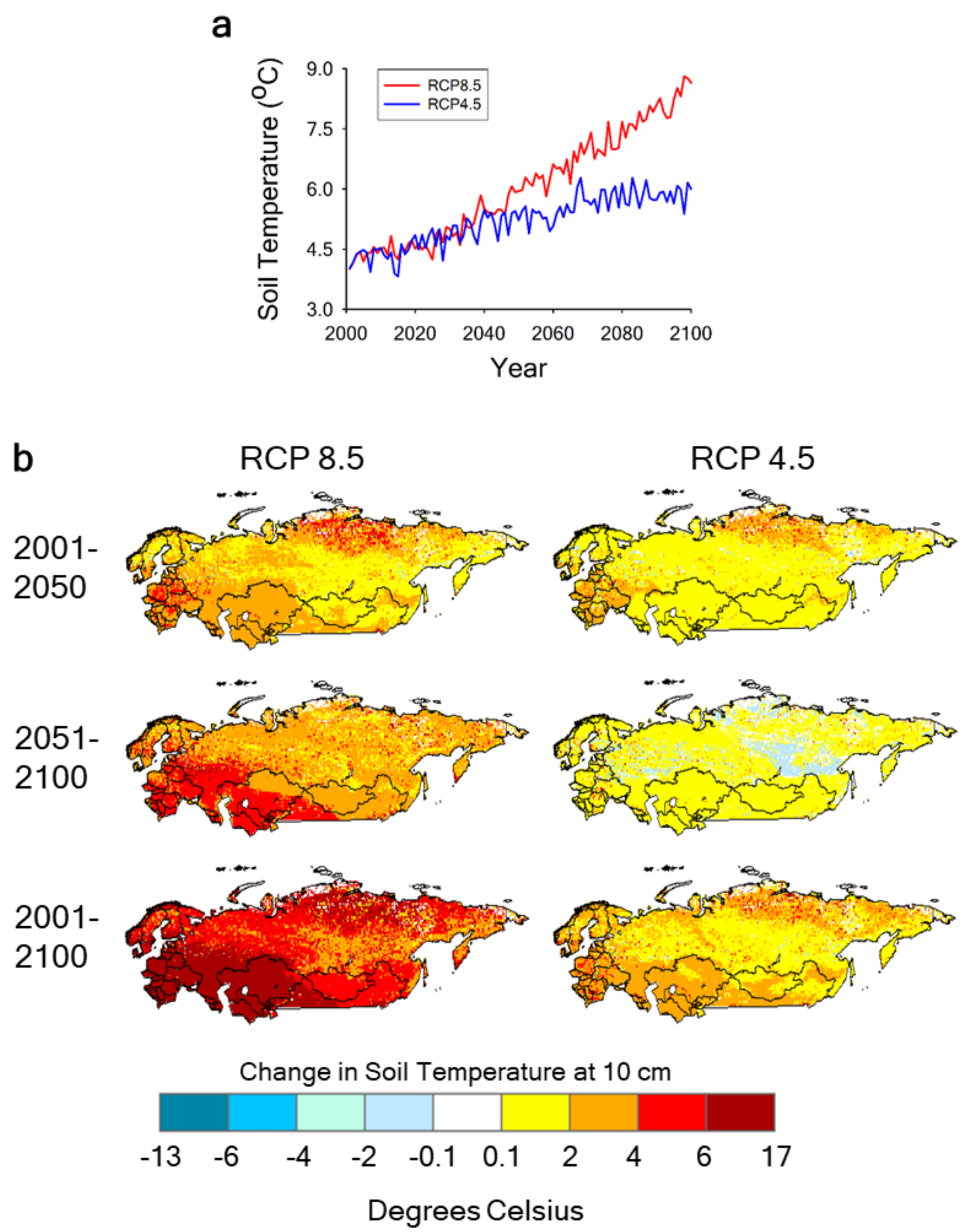

Supplementary Figure 3. Projected changes in soil temperature at 10 cm depth across Northern Eurasia during the 21st century. **a** Temporal variations in regional mean soil temperature. **b** Spatial variations in soil temperature change during the first half (2001-2050) and second half (2051-2100) of the 21st century along with the entire study period (2001-2100) as projected by the RCP8.5 and RCP4.5 global change scenarios.

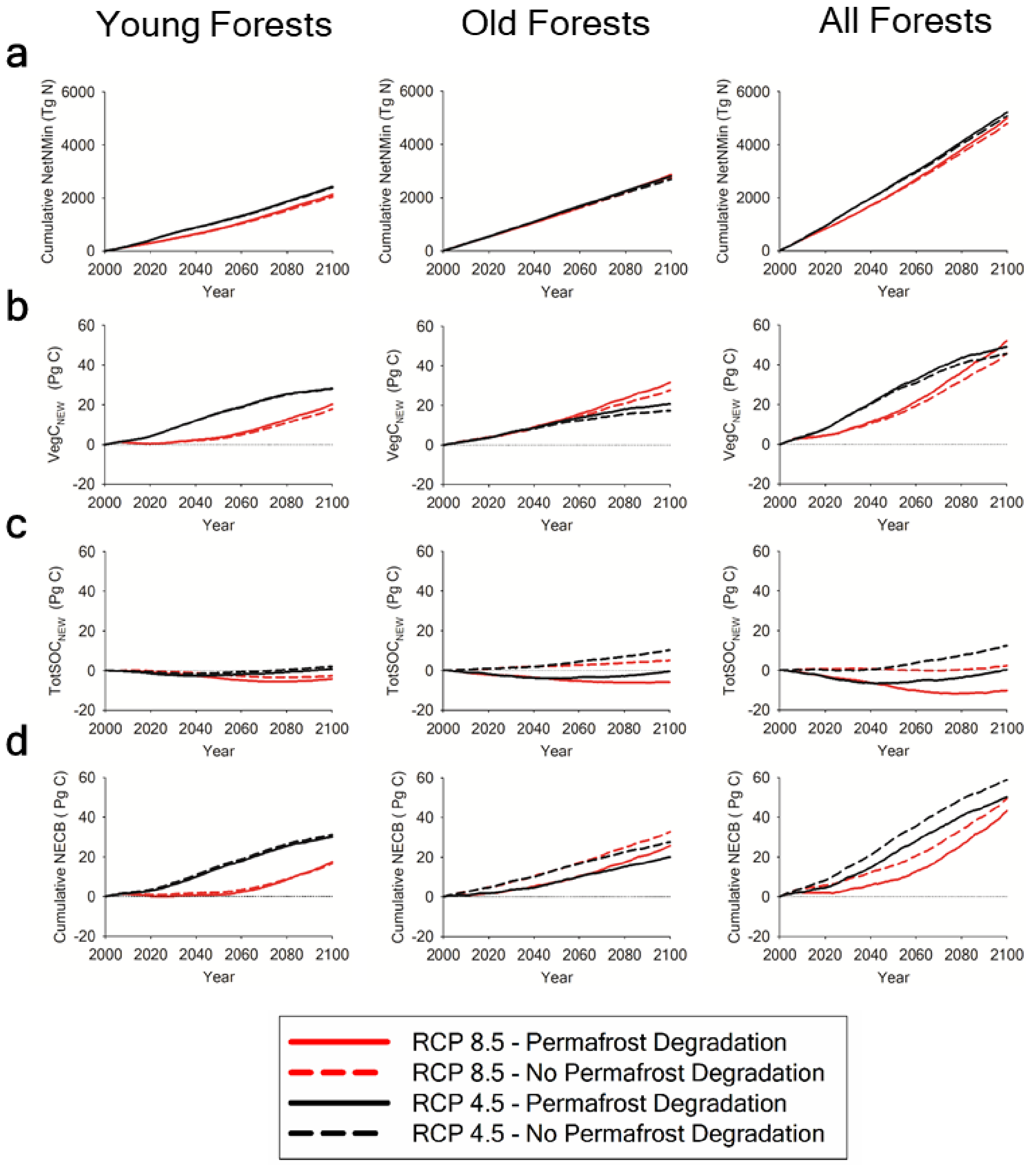

Supplementary Figure 4. Influence of stand age and permafrost degradation on the forest responses of N availability and C sequestration to the RCP8.5 and RCP4.5 global change scenarios over the 21st century. **a** Cumulative net N mineralization (NetNMin). **b** Cumulative change in new-carbon sequestered by forest vegetation (VegC$_{NEW}$), **c** Cumulative change in new-carbon sequestered/lost by forest soil organic matter (TotSOC$_{NEW}$). **d** Cumulative carbon sequestration or loss as represented by net ecosystem carbon balance (NECB). Positive values represent C or N gains by the ecosystem and negative values represent losses. Young forests are all stands that are less than 120-years old. Old forests are all stands that are 120-years or older.

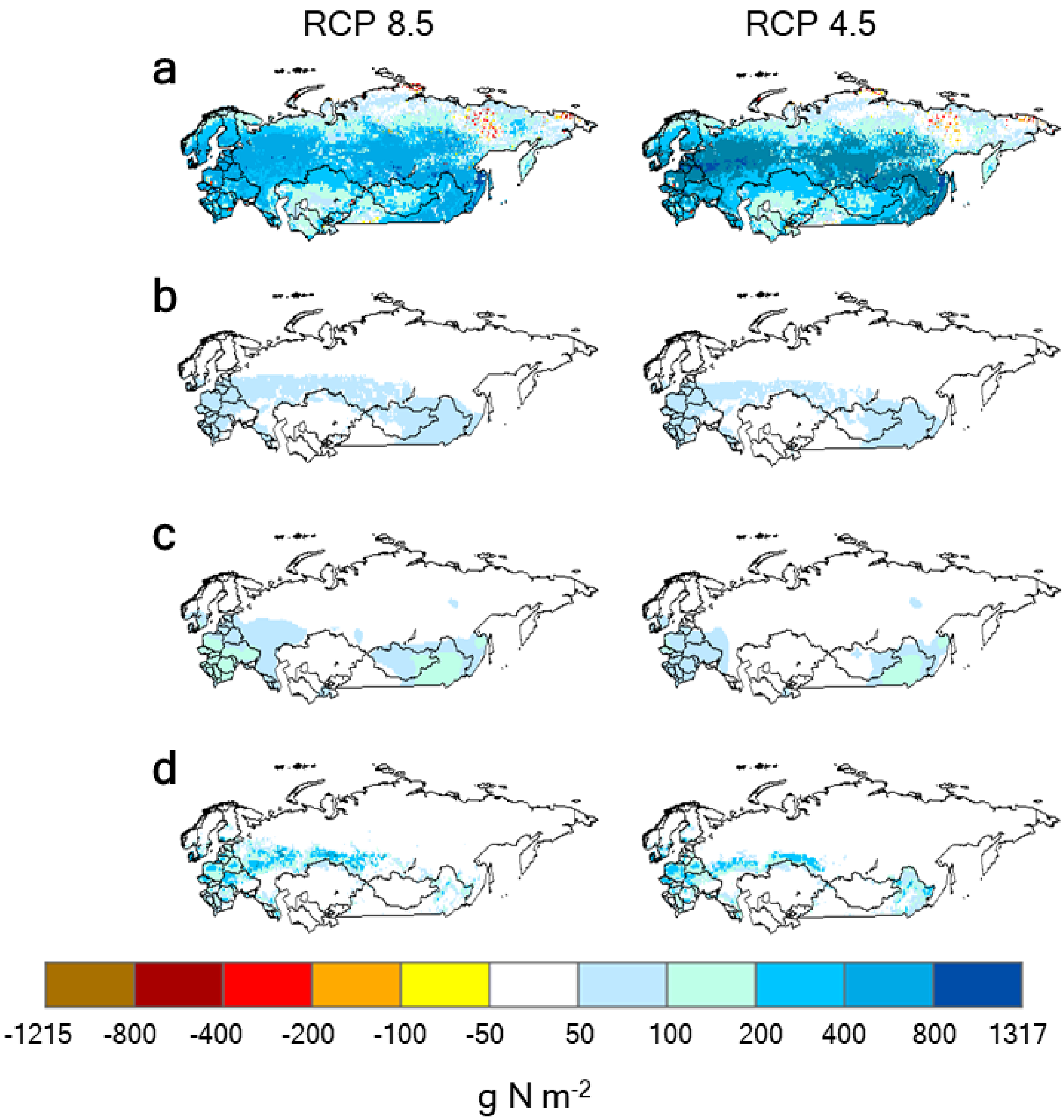

Supplementary Figure 5. Projected spatial patterns of cumulative nitrogen inputs across all land ecosystems in Northern Eurasia under the RCP8.5 and RCP4.5 global change scenarios during the 21st century. **a** Net N mineralization. **b** Biological N fixation. **c** Atmospheric N deposition. **d** N fertilizer applications.

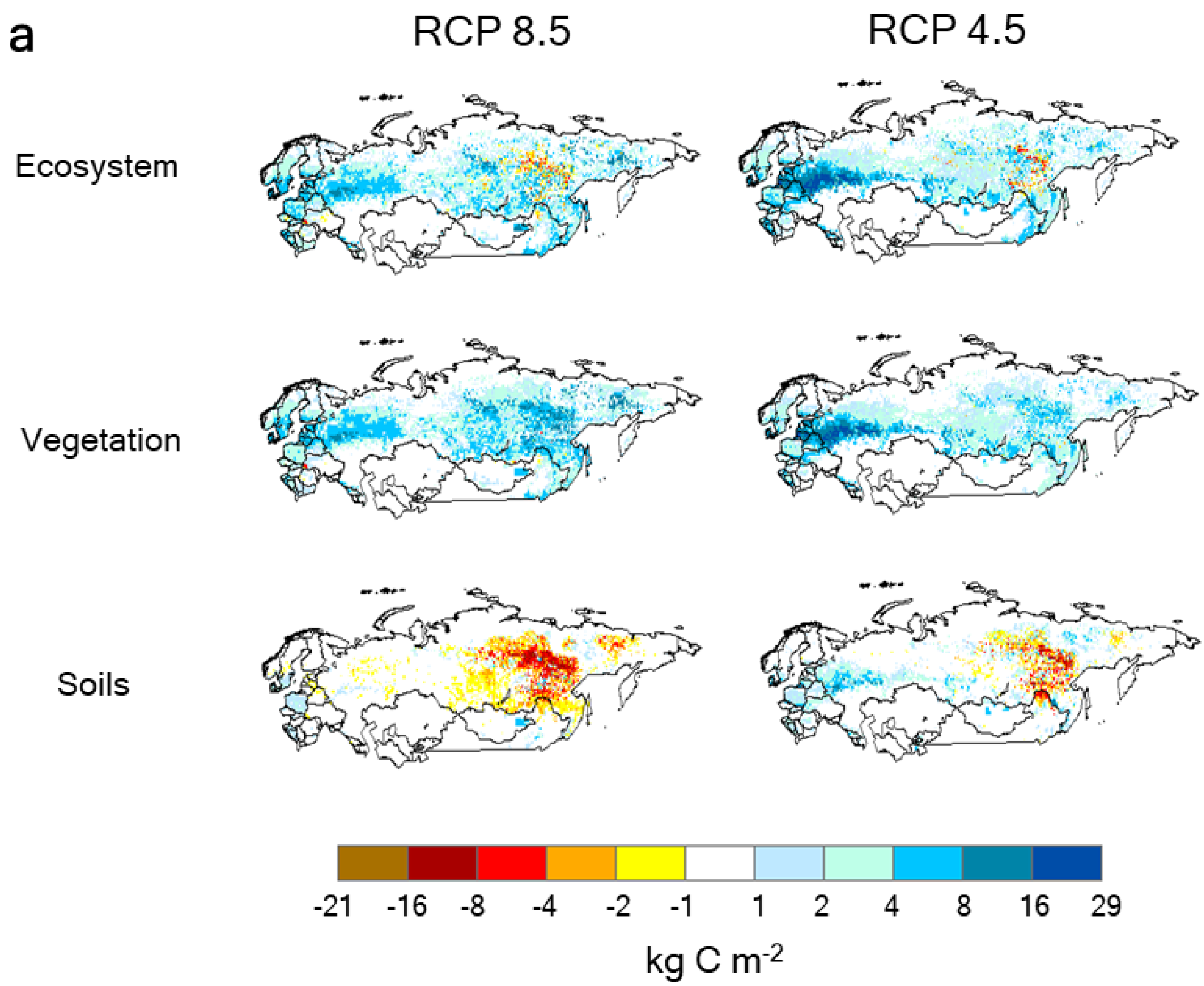

Supplementary Figure 6. Evolution of C sequestered/lost by vegetation and soils in forest ecosystems across Northern Eurasia over the 21st century projected under the RCP8.5 and RCP4.5 global change scenarios. Positive values represent C sinks and negative values represent C sources. **a** Entire study period (2001-2100). **b** First half of the 21st century (2001-2050). **c** Second half of the 21st century (2051-2100).

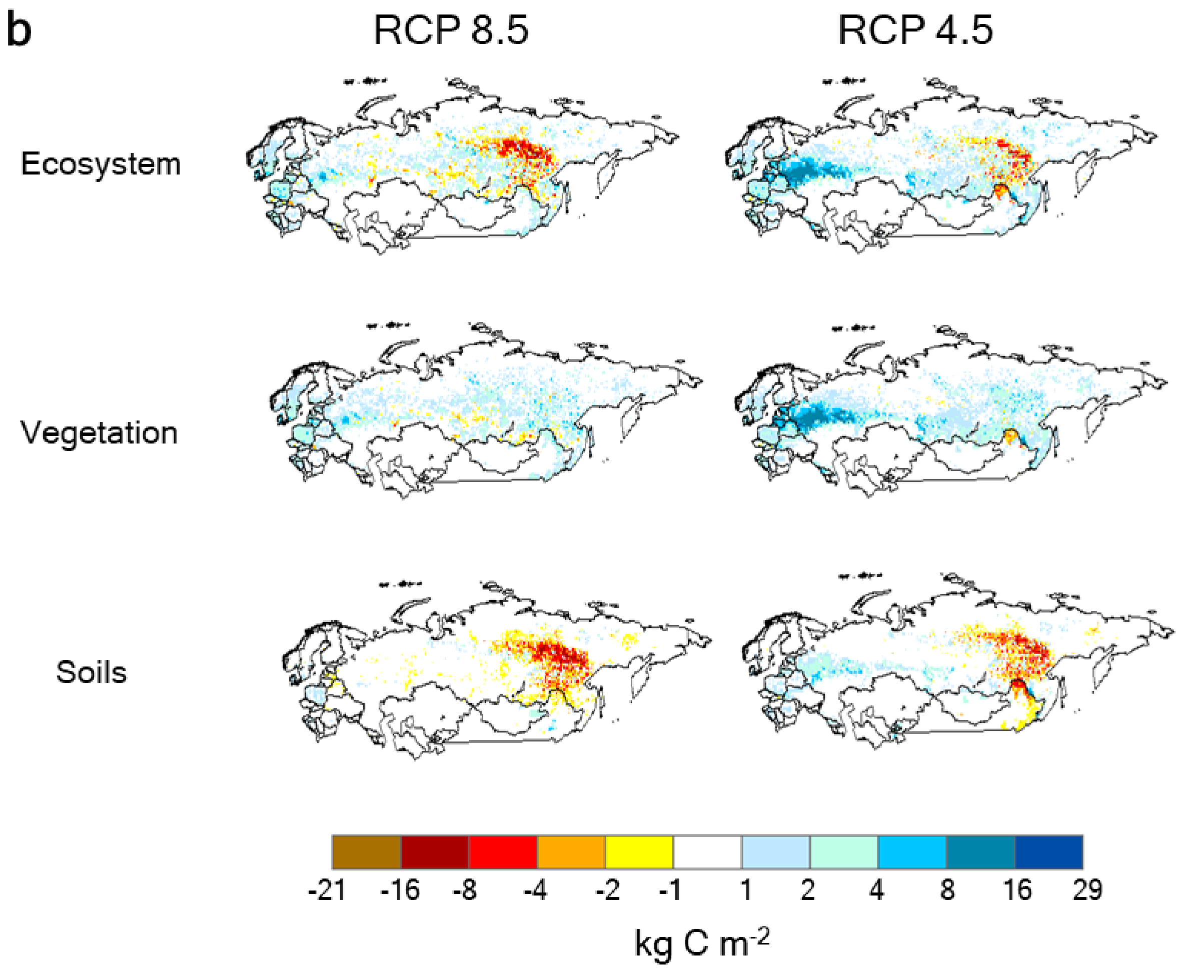

Supplementary Figure 6 (continued). Evolution of C sequestered/lost by vegetation and soils in forest ecosystems across Northern Eurasia over the 21st century projected under the RCP8.5 and RCP4.5 global change scenarios. Positive values represent C sinks and negative values represent C sources. **a** Entire study period (2001-2100). **b** First half of the 21st century (2001-2050). **c** Second half of the 21st century (2051-2100).

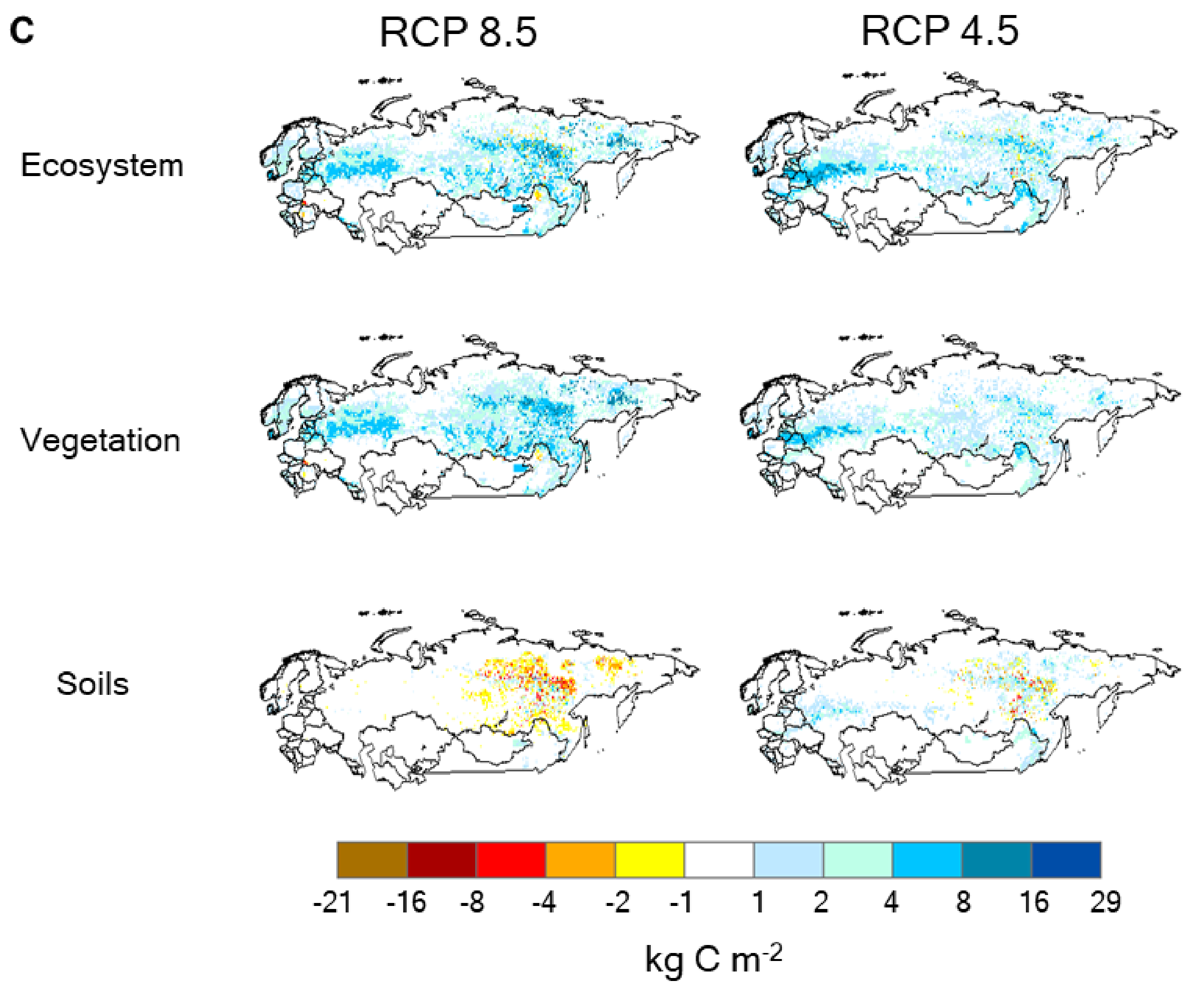

Supplementary Figure 6 (continued). Evolution of C sequestered/lost by vegetation and soils in forest ecosystems across Northern Eurasia over the 21st century projected under the RCP8.5 and RCP4.5 global change scenarios. Positive values represent C sinks and negative values represent C sources. **a** Entire study period (2001-2100). **b** First half of the 21st century (2001-2050). **c** Second half of the 21st century (2051-2100).

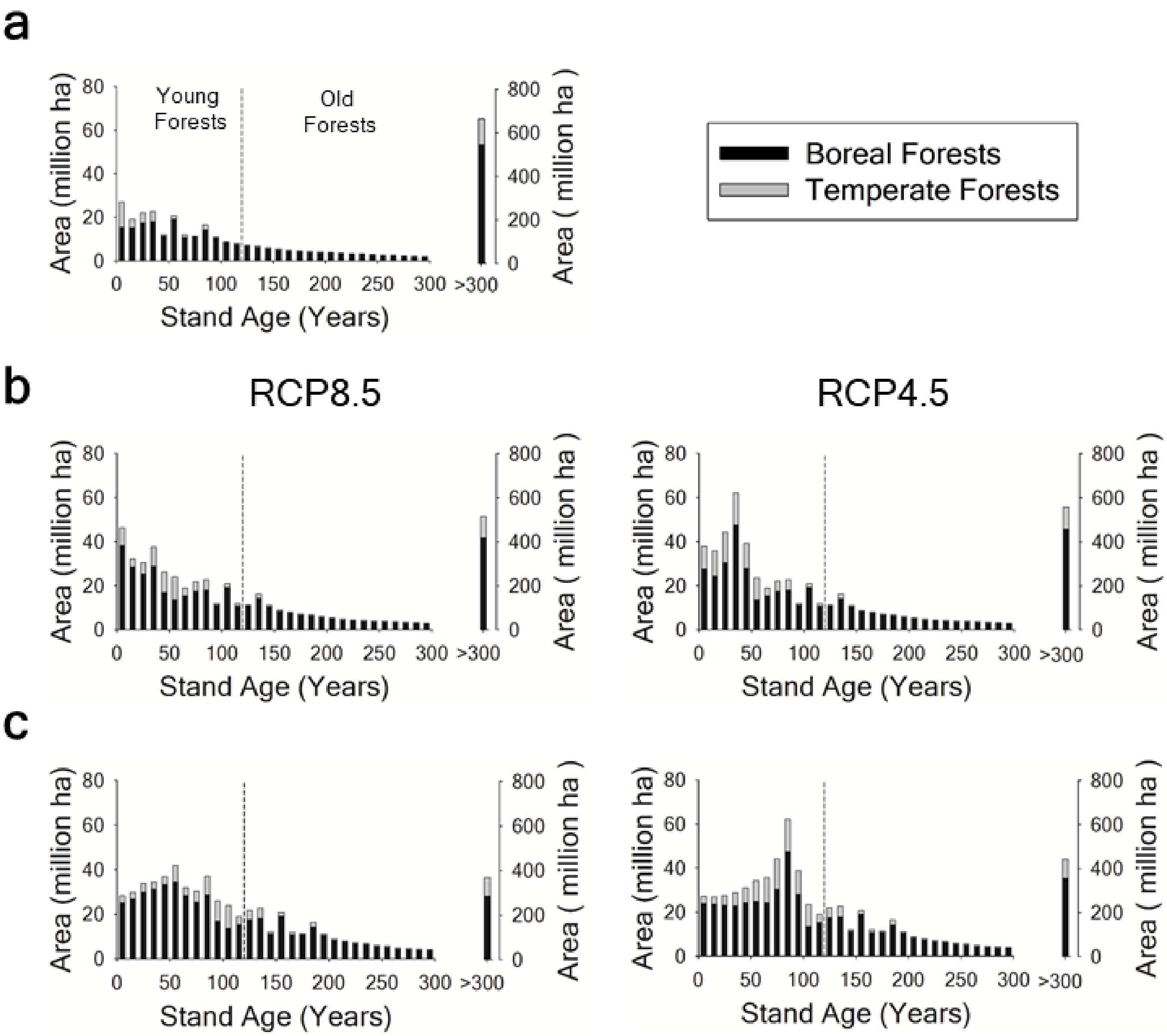

Supplementary Figure 7. Projected changes in stand age distribution of all forests over the 21st century under the RCP8.5 and RCP4.5 global change scenarios. Forests are stratified into boreal (black bars) and temperate (gray bars) forests. **a** Stand age distribution during year 2000. **b** Stand age distributions during year 2050. **c** Stand age distributions during year 2100. The stand age distribution in 2000 is the same for both global change scenarios.

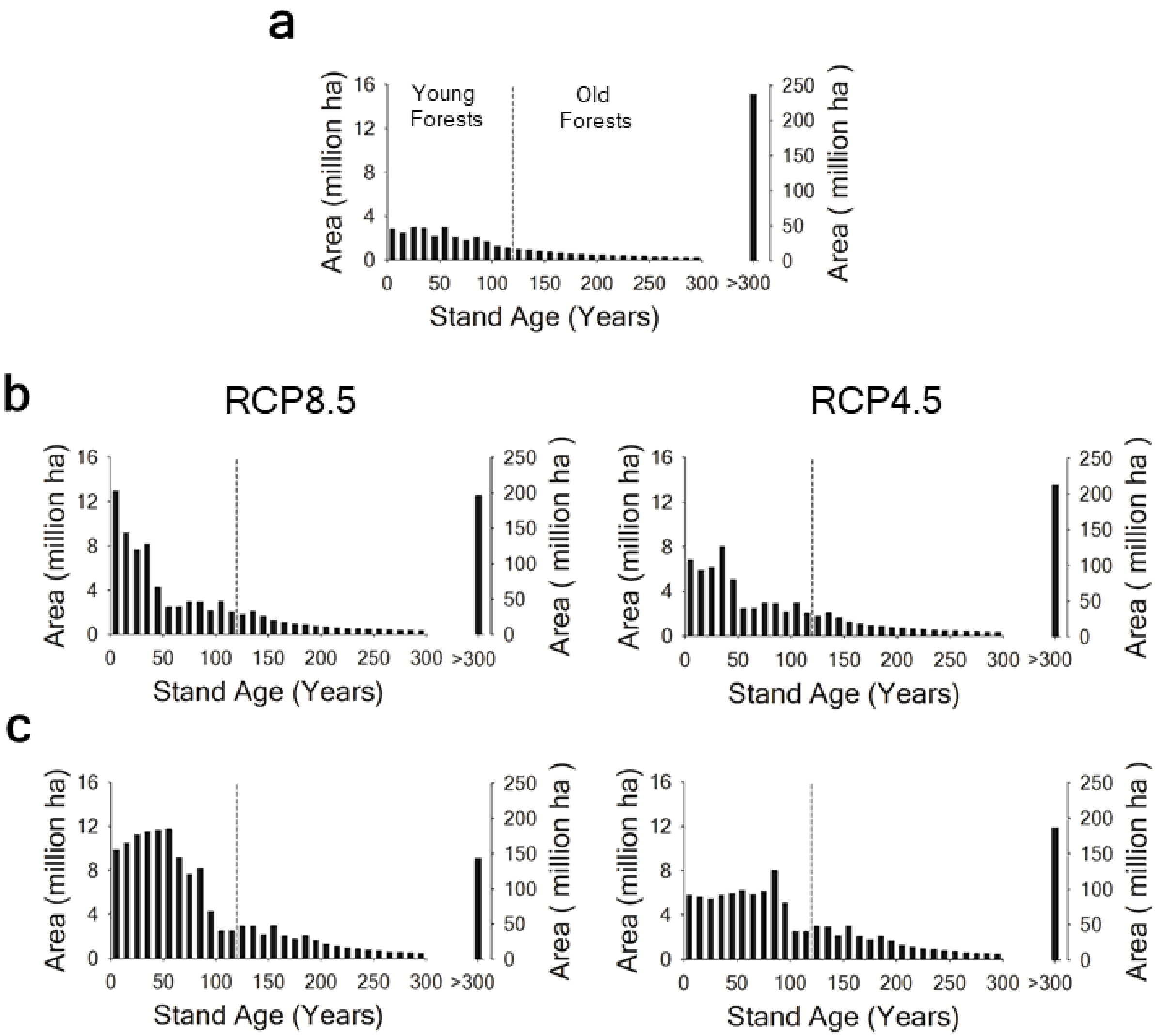

Supplementary Figure 8. Projected changes in stand age distribution of permafrost forests over the 21st century under the RCP8.5 and RCP4.5 global change scenarios. Permafrost forests are those stands underlain by permafrost during 2000. **a** Stand age distribution during year 2000. **b** Stand age distributions during year 2050. **c** Stand age distributions during year 2100. The stand age distribution in 2000 is the same for both global change scenarios.

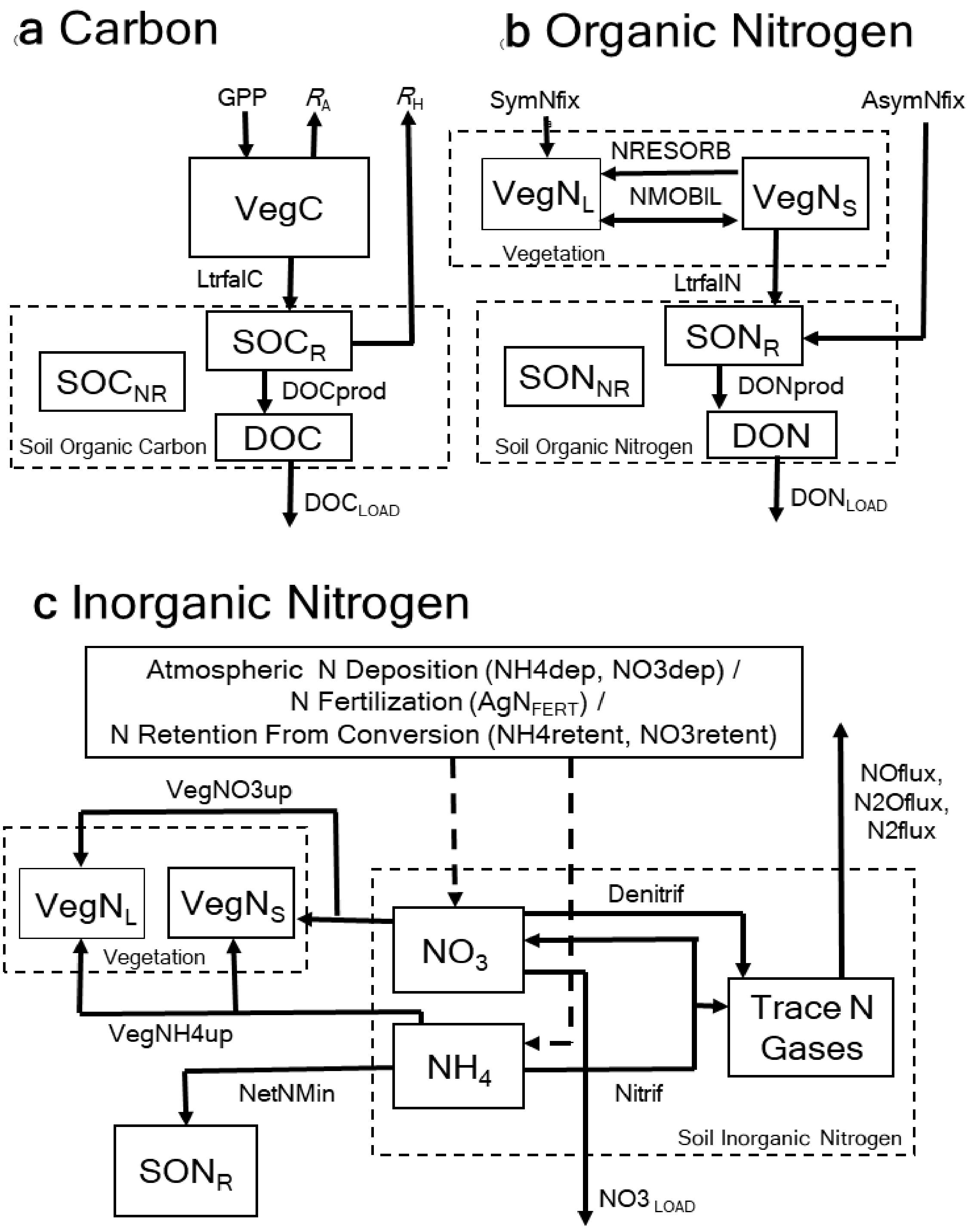
a Carbon
GPP
RA
RH
VegC
LtrfalC
SOCR
SOCNR
DOCprod
DOC
Soil Organic Carbon
DOCLOAD
b Organic Nitrogen
SymNfix
AsymNfix
NRESORB
VegNL
NMOBIL
VegNS
Vegetation
LtrfalN
SONR
SONNR
DONprod
DON
Soil Organic Nitrogen
DONLOAD
c Inorganic Nitrogen
Atmospheric N Deposition (NH4dep, NO3dep) /
N Fertilization (AgNFERT) /
N Retention From Conversion (NH4retent, NO3retent)
VegNO3up
NOflux,
N2Oflux,
N2flux
VegNL
VegNS
Vegetation
Denitrif
NO3
Trace N
Gases
VegNH4up
NH4
NetNMin
Nitrif
Soil Inorganic Nitrogen
SONR
NO3 LOAD

Supplementary Figure 9. Structure of the Terrestrial Ecosystem Model for natural ecosystems. **a** Carbon pools include vegetation carbon (VegC), reactive soil organic carbon (SOC_R), non-reactive soil organic carbon (SOC_{NR}) and dissolved organic carbon (DOC). Carbon fluxes include gross primary production (GPP), autotrophic respiration (R_A), heterotrophic respiration (R_H), litterfall carbon (LtrfalC), production of dissolved organic carbon (DOCprod) and terrestrial DOC loading of neighboring river networks (DOC_{LOAD}) into and from carbon pools that include. **b** Organic nitrogen pools include vegetation structural nitrogen ($VegN_S$), vegetation labile nitrogen ($VegN_L$), reactive soil organic nitrogen (SON_R), non-reactive soil organic nitrogen (SON_{NR}) and dissolved organic nitrogen (DON). Organic nitrogen fluxes include symbiotic (SymNfix) and asymbiotic (AsymNfix) nitrogen fixation, N resorption from dying tissue into $VegN_S$ (NRESORB), N mobilized between $VegN_S$ and $VegN_L$ (NMOBIL), litterfall nitrogen (LtrfalN), production of dissolved organic nitrogen (DONprod) and terrestrial DON loading of neighboring river networks (DON_{LOAD}). **c** Inorganic nitrogen pools include soil ammonium (NH_4), nitrate (NO_3), and the trace N gases of nitric oxide (NO), nitrous oxide (N_2O) and dinitrogen (N_2). Inorganic nitrogen fluxes include atmospheric deposition inputs of ammonium (NH4dep) and nitrate (NO3dep), agricultural application of nitrogen fertilizers (AgN_{FERT}), inputs of ammonium (NH4retent) and nitrate (NO3retent) retained by the ecosystem during the combustion of slash following agricultural conversion or timber harvest, vegetation uptake of ammonium (VegNH4up) and nitrate (VegNO3up), net nitrogen mineralization (NetNMin), nitrification (Nitrif), denitrification (Denitrif), terrestrial nitrate loading of neighboring river networks ($NO3_{LOAD}$), trace gas emissions of nitric oxide (NOflux), nitrous oxide (N2Oflux), and dinitrogen (N2flux).

Supplementary Table 1a. Estimates of carbon stocks in vegetation and soils of Northern Eurasia[a]

Source	Region	Biome	Year	Vegetation C (Pg C)	Soil C (Pg C)	Total C (Pg C)
This study	*Northern Eurasia*	*All*<br>*Forest*	*2000*<br>*2000*	*109*<br>*91*	*374 (1-2 m)[d]*<br>*139 (2 m)*	*483*<br>*230*
FAO[1]	Northern Eurasia[b] except China	Forest<br>Forest	2000<br>2015	39<br>41	N/A<br>107	N/A<br>148
Goodale *et al.*[2]	Russia & Europe	Forest	1990	42	172 (1 m)	214
Pan *et al.*[3]	Russia & boreal Europe	Forest	2000	38	175[e] (1 m)	213
Thurner et al.[4]	Russia & Europe	Forest	N/A	51.1±19.1	N/A	N/A
Tarnocai *et al.*[5]	Eurasian NCPR[c]	All	N/A	N/A	331 (1 m)	N/A
Stolbovoi[6]	Russia	All	N/A	N/A	297 (1 m)<br>373 (2 m)	N/A

[a] region, biome and year of inventory listed when available
[b]see Supplementary Table 1b; vegetation carbon, soil carbon, and total carbon for 2015 are all underestimated as a result of missing data for some components in some countries
[c]Northern Circumpolar Permafrost Region (NCPR)
[d]Forests assume a rooting depth of about 2 meters whereas non-forests assume a rooting depth of about 1 meter
[e]Live fine roots may be included if excluded from vegetation carbon

Supplementary Table 1b. FAO estimates[1] of vegetation, soil (dead wood, litter, and soil organic matter) and total carbon stocks in forests of countries within Northern Eurasia

Country	Veg C in 2000 (Pg C)	Veg C in 2015 (Pg C)	Soil C in 2015 (Pg C)	Total C in 2015 (Pg C)
Albania	0.049	0.050	0.094	0.144
Armenia	0.016	0.016	0.021[a]	0.037
Belarus	0.482	0.646	0.840	1.486
Bosnia	0.118	0.118	N/A	0.118
Bulgaria	0.161	0.213	0.371[a]	0.584
Croatia	0.221	0.256	N/A	0.256
Czech Republic	0.323	0.366	0.205	0.571
Denmark	0.037	0.041	0.111	0.152
Estonia	0.158	0.165	0.354[b]	0.519
Finland	0.716	0.780	4.324	5.104
Georgia	0.203	0.212	0.245[a]	0.457
Hungary	0.107	0.122	0.005[c]	0.127
Kazakhstan	0.137	0.137	N/A	0.137
Kyrgyzstan	0.034	0.017	0.097	0.114
Latvia	0.231	0.285	0.343	0.628
Lithuania	0.146	0.167	0.220	0.387
Mongolia	0.626	N/A	N/A	N/A
Montenegro	0.033	0.056	0.004[c]	0.060
Norway	0.377	0.476	N/A	0.476
Poland	0.546	0.822	0.032[c]	0.854
Romania	0.383	0.616	0.765	1.381
Russia	32.157	32.800	95.100	127.900
Serbia	0.138	0.237	0.335	0.572
Slovakia	0.190	0.218	0.309	0.527
Slovenia	0.107	0.141	0.148	0.289
Sweden	1.016	1.114	2.561	3.675
Tajikistan	0.003	0.003	0.032[b]	0.035
Turkmenistan	0.011	0.313	0.079[d]	0.392
Ukraine	0.662	0.783	N/A	0.783
Uzbekistan	0.014	0.042	0.012	0.054
Northern Eurasia	39.353	41.212	106.607	147.819

[a]Missing value for dead wood
[b]Missing value for litter
[c]Missing values for litter and soil organic matter
[d]Missing values for dead wood and litter

Supplementary Table 2. Estimates of carbon sequestration in Northern Eurasia

Approach	Region	Period	Biome	Net C Gain (Pg C yr^{-1})	Source
Land Carbon Modeling:					
TEM	Northern Eurasia	2001-2005	All<br>Forest	0.34<br>0.28	This Study
8 DGVMs[a]	Russia	1988-2008	All	0.02-0.35	Dolman et al.[7]
Inventory:					
	Russia and boreal Europe	2000-2007	Forest	0.49	Pan et al.[3]
	Russia	1961-1999	Forest	0.32-0.43	Hayes et al.[8]
LEA[b]	Russia	2009	All<br>Forest	0.76<br>0.69	Dolman et al.[7]
	Russia	1998-2010	Forest	0.24-0.55	Schaphoff et al.[9]
Eddy Covariance:					
	Russia	1990-2008	All	0.34-0.69	Dolman et al.[7]
Inverse Modeling:					
	Boreal Eurasia	2000-2009	All	0.35-0.56	Saeki et al.[10]
	Russia	1990=2008	All	0.35-1.35	Dolman et al.[7]

[a]Dynamic Global Vegetation Model (DGVM)
[b]Land Ecosystem Assessment (LEA)

Supplementary Table 3. Area changes in land cover/land use (million ha) in Northern Eurasia projected over the 21st century under two climate change projections (RCP8.5, RCP4.5) and land-use transitions described by Hurtt et al.[11]

Land Cover	2000	RCP8.5		RCP4.5	
		2050	2100	2050	2100
Croplands	289	278	277	197	169
Pastures	618	606	614	486	491
Young Forests					
Boreal	158	242	308	263	301
Temperate	33	62	63	87	096
All	191	304	371	350	397
Old Forests					
Boreal	617	535	467	574	538
Temperate	130	109	109	111	112
All	747	644	576	685	650
All Forests					
Boreal	775	777	775	837	839
Temperate	163	171	172	198	208
All	938	948	947	1,035	1,047
Temperate Grasslands	99	111	108	174	184
Temperate Shrublands	129	130	128	174	174
Tundra	425	425	425	426	426
Wetlands	183	183	182	188	189
Deserts	160	160	160	161	161
Lakes	19	19	19	19	19
Glaciers	8	8	8	8	8
Total	2,868	2,868	2,868	2,868	2,868

Supplementary Table 4. Area changes in land cover/land use (million ha) for areas underlain by permafrost over the 21st century under two climate change projections (RCP8.5, RCP4.5) and the land-use transitions described by Hurtt et al.[11]

Land Cover	2000	RCP8.5		RCP4.5	
		2050	2100	2050	2100
Croplands	34	25	10	21	17
Pastures	117	83	48	73	64
Young Forests					
Boreal	25	24	14	24	20
Temperate	1	2	1	4	3
All	26	26	15	28	23
Old Forests					
Boreal	238	123	31	163	130
Temperate	9	3	1	4	2
All	247	126	32	167	132
All Forests					
Boreal	263	147	45	187	150
Temperate	10	5	2	8	5
All	273	152	47	195	155
Temperate Grasslands	33	30	15	47	46
Temperate Shrublands	2	2	0	4	3
Tundra	287	233	97	255	228
Wetlands	42	31	16	35	31
Deserts	127	118	91	120	116
Lakes	5	4	2	5	4
Glaciers	0	0	0	0	0
Total	920	678	326	755	664

Supplementary Table 5. Comparison of cumulative net ecosystem carbon balance (NECB, Pg C) among land cover types in Northern Eurasia over different time periods under two climate change projections (RCP8.5, RCP4.5)

Land Cover	RCP8.5			RCP4.5		
	2001-2050	2051-2100	2001-2100	2001-2050	2051-2100	2001-2100
Croplands	-2.0	-1.7	-3.7	-0.5	-0.5	-1.0
Pastures	-1.6	-1.3	-2.9	-0.5	-0.6	-1.1
Young Forests						
Boreal	-2.0	13.3	11.3	9.5	9.7	19.2
Temperate	2.8	3.4	6.2	4.8	6.1	10.9
All	0.8	16.7	17.5	14.3	15.8	30.1
Old Forests						
Boreal	5.2	15.5	20.7	5.5	11.5	17.0
Temperate	2.3	2.7	5.0	2.1	1.0	3.1
All	7.5	18.2	25.7	7.6	12.5	20.1
Forests						
Boreal	3.2	28.8	32.0	15.0	21.2	36.2
Temperate	5.1	6.1	11.2	6.9	7.1	14.0
All	8.3	34.9	43.2	21.9	28.3	50.2
Temperate Grasslands	0.2	0.1	0.3	0.4	0.1	0.5
Temperate Shrublands	0.5	0.7	1.2	0.2	0.2	0.4
Tundra	0.3	-2.9	-2.6	0.4	-0.1	0.3
Wetlands	0.7	2.3	3.0	1.5	1.2	2.7
Deserts	0.9	1.2	2.1	0.9	0.8	1.7
Total	7.3	33.3	40.6	24.3	29.4	53.7

Supplementary Table 6. Comparison of cumulative carbon sequestration in vegetation ($VegC_{NEW}$, Pg C) among land cover types in Northern Eurasia over different time periods under two climate change projections (RCP8.5, RCP4.5)

Land Cover	RCP8.5			RCP4.5		
	2001-2050	2051-2100	2001-2100	2001-2050	2051-2100	2001-2100
Croplands	0.1	-0.1	0.0	-0.1	-0.1	-0.2
Pastures	0.0	0.1	0.1	0.0	0.0	0.0
Young Forests						
Boreal	1.1	14.5	15.6	11.3	8.6	19.9
Temperate	2.4	2.3	4.7	4.6	3.8	8.4
All	3.5	16.8	20.3	15.9	12.4	28.3
Old Forests						
Boreal	9.9	17.4	27.3	9.6	8.6	18.2
Temperate	2.2	2.1	4.3	1.9	0.6	2.5
All	12.1	19.5	31.6	11.5	9.2	20.7
Forests						
Boreal	11.0	31.9	42.9	20.9	17.2	38.1
Temperate	4.6	4.4	9.0	6.5	4.4	10.9
All	15.6	36.3	51.9	27.4	21.6	49.0
Temperate Grasslands	0.1	0.1	0.2	0.5	0.1	0.6
Temperate Shrublands	0.1	0.2	0.3	0.3	0.1	0.4
Tundra	0.8	0.9	1.7	0.6	0.4	1.0
Wetlands	1.2	3.1	4.3	1.9	1.0	2.9
Deserts	0.3	0.3	0.6	0.2	0.1	0.3
Total	18.2	40.9	59.1	30.8	23.2	54.0

Supplementary Table 7. Comparison of cumulative carbon sequestration in soil organic matter ($TotSOC_{NEW}$, Pg C) among land cover types in Northern Eurasia over different time periods under two climate change projections (RCP8.5, RCP4.5)

Land Cover	RCP8.5			RCP4.5		
	2001-2050	2051-2100	2001-2100	2001-2050	2051-2100	2001-2100
Croplands	-1.9	-1.5	-3.4	0.0	-0.4	-0.4
Pastures	-1.3	-1.3	-2.6	-0.5	-0.4	-0.9
Young Forests						
Boreal	-4.1	-1.5	-5.6	-2.3	0.7	-1.6
Temperate	0.2	1.1	1.3	-0.1	2.5	2.4
All	-3.9	-0.4	-4.3	-2.4	3.2	0.8
Old Forests						
Boreal	-4.8	-1.8	-6.6	-4.1	3.0	-1.1
Temperate	0.3	0.4	0.7	0.2	0.3	0.5
All	-4.5	-1.4	-5.9	-3.9	3.3	-0.6
Forests						
Boreal	-8.9	-3.3	-12.2	-6.4	3.7	-2.7
Temperate	0.5	1.5	2.0	0.1	2.8	2.9
All	-8.4	-1.8	-10.2	-6.3	6.5	0.2
Temperate Grasslands	0.1	0.0	0.1	-0.1	0.0	-0.1
Temperate Shrublands	0.4	0.5	0.9	-0.1	0.1	0.0
Tundra	-0.5	-3.8	-4.3	-0.2	-0.5	-0.7
Wetlands	-0.6	-0.9	-1.5	-0.4	-0.1	-0.5
Deserts	0.6	0.9	1.5	0.7	0.7	1.4
Total	-11.6	-7.9	-19.5	-6.9	5.9	-1.0

Supplementary Table 8. Comparison of total nitrogen availability (Tg N) among land cover types in Northern Eurasia over different time periods under two climate change projections (RCP8.5, RCP4.5)

Land Cover	RCP8.5			RCP4.5		
	2001-2050	2051-2100	2001-2100	2001-2050	2051-2100	2001-2100
Croplands	1,021.0	1,115.1	2,136.1	839.0	710.2	1,549.2
Pastures	877.2	1,027.6	1,904.8	770.3	774.4	1,544.7
Young Forests						
Boreal	758.1	1,246.9	2,005.0	951.7	1,158.8	2,110.5
Temperate	188.6	231.1	419.7	281.1	330.3	611.4
All	946.7	1,478.0	2,424.7	1,232.8	1,489.1	2,721.9
Old Forests						
Boreal	1,224.3	1,364.0	2,588.3	1,270.4	1,284.9	2,555.3
Temperate	393.1	403.7	796.8	388.2	333.8	722.0
All	1,617.4	1,767.7	3,385.1	1,658.6	1,618.7	3,277.3
Forests						
Boreal	1,982.4	2,610.9	4,593.3	2,222.1	2,443.7	4,665.8
Temperate	581.7	634.8	1,216.5	669.3	664.1	1,333.4
All	2,564.1	3,245.7	5,809.8	2,891.4	3,107.8	5,999.2
Temperate Grasslands	199.0	238.8	437.8	257.6	379.2	636.8
Temperate Shrublands	147.5	152.8	300.3	205.6	250.0	455.6
Tundra	214.5	246.3	460.8	211.3	229.8	441.1
Wetlands	329.9	407.4	737.3	346.3	347.9	694.2
Deserts	45.4	54.3	99.7	46.4	45.0	91.4
Total	5,398.6	6,488.0	11,886.6	5,567.9	5,844.3	11,412.2

Supplementary Table 9. Comparison of cumulative net nitrogen mineralization (NetNMin, Tg N) among land cover types in Northern Eurasia over different time periods under two climate change projections (RCP8.5, RCP4.5)

Land Cover	RCP8.5			RCP4.5		
	2001-2050	2051-2100	2001-2100	2001-2050	2051-2100	2001-2100
Croplands	221.8	506.2	728.0	136.7	295.6	432.3
Pastures	548.9	678.0	1,226.9	505.6	552.6	1,058.2
Young Forests						
Boreal	679.5	1,125.4	1,804.9	865.6	1,050.4	1,916.0
Temperate	149.9	178.5	328.4	239.0	273.4	512.4
All	829.4	1,303.9	2,133.3	1,104.6	1,323.8	2,428.4
Old Forests						
Boreal	1,040.3	1,191.9	2,232.2	1,090.1	1,127.1	2,217.2
Temperate	305.3	319.9	625.2	309.4	272.3	581.7
All	1,345.6	1,511.8	2,857.4	1,399.5	1,399.4	2,798.9
Forests						
Boreal	1,719.8	2,317.3	4,037.1	1,955.7	2,177.5	4,133.2
Temperate	455.2	498.4	953.6	548.4	545.7	1,094.1
All	2,175.0	2,815.7	4,990.7	2,504.1	2,723.2	5,227.3
Temperate Grasslands	142.2	168.4	310.6	194.5	302.0	496.5
Temperate Shrublands	105.2	109.2	214.4	160.9	201.1	362.0
Tundra	143.2	162.0	305.2	143.2	160.9	304.1
Wetlands	268.8	339.5	608.3	288.2	291.5	579.7
Deserts	18.5	23.3	41.8	21.1	20.2	41.3
Total	3,623.6	4,802.3	8,425.9	3,954.3	4,547.1	8,501.4

Supplementary Table 10. Comparison of cumulative biological nitrogen fixation (BiolNfix, Tg N) among land cover types in Northern Eurasia over different time periods under two climate change projections (RCP8.5, RCP4.5)

Land Cover	RCP8.5			RCP4.5		
	2001-2050	2051-2100	2001-2100	2001-2050	2051-2100	2001-2100
Croplands	74.6	80.0	154.6	64.9	52.3	117.2
Pastures	141.5	155.2	296.7	125.3	118.8	244.1
Young Forests						
Boreal	39.9	68.5	108.4	46.8	66.7	113.5
Temperate	14.7	20.8	35.5	19.0	28.8	47.8
All	54.6	89.3	143.9	65.8	95.5	161.3
Old Forests						
Boreal	94.4	99.0	193.4	97.7	96.6	194.3
Temperate	32.8	31.8	64.6	33.4	30.3	63.7
All	127.2	130.8	258.0	131.1	126.9	258.0
Forests						
Boreal	134.3	167.5	301.8	144.5	163.3	307.8
Temperate	47.5	52.6	100.1	52.4	59.1	111.5
All	181.8	220.1	401.9	196.9	222.4	419.3
Temperate Grasslands	23.5	29.3	52.8	30.2	42.6	72.8
Temperate Shrublands	21.1	21.5	42.6	24.1	29.0	53.1
Tundra	38.6	52.1	90.7	38.3	43.3	81.6
Wetlands	31.0	38.4	69.4	31.5	35.2	66.7
Deserts	13.8	17.5	31.3	13.8	15.1	28.9
Total	525.9	614.1	1,140.0	525.0	558.7	1,083.7

Supplementary Table 11. Comparison of cumulative total atmospheric nitrogen deposition (TotNdep, Tg N) among land cover types in Northern Eurasia over different time periods under two climate change projections (RCP8.5, RCP4.5)

Land Cover	RCP8.5			RCP4.5		
	2001-2050	2051-2100	2001-2100	2001-2050	2051-2100	2001-2100
Croplands	112.3	116.3	228.6	81.9	53.1	135.0
Pastures	186.8	194.4	381.2	139.4	103.0	242.4
Young Forests						
Boreal	38.7	53.0	91.7	39.3	41.7	81.0
Temperate	24.0	31.8	55.8	23.1	28.1	51.2
All	62.7	84.8	147.5	62.4	69.8	132.2
Old Forests						
Boreal	89.6	73.1	162.7	82.6	61.2	143.8
Temperate	55.0	52.0	107.0	45.4	31.2	76.6
All	144.6	125.1	269.7	128.0	92.4	220.4
Forests						
Boreal	128.3	126.1	254.4	121.9	102.9	224.8
Temperate	79.0	83.8	162.8	68.5	59.3	127.8
All	207.3	209.9	417.2	190.4	162.2	352.6
Temperate Grasslands	33.3	41.1	74.4	32.9	34.6	67.5
Temperate Shrublands	21.2	22.1	43.3	20.6	19.9	40.5
Tundra	32.7	32.2	64.9	29.8	25.6	55.4
Wetlands	30.1	29.5	59.6	26.6	21.2	47.8
Deserts	13.1	13.5	26.6	11.5	9.7	21.2
Total	636.8	659.0	1,295.8	533.1	429.3	962.4

Supplementary Table 12. Comparison of cumulative agricultural nitrogen fertilization (AgFertN, Tg N) among land cover types in Northern Eurasia over different time periods under two climate change projections (RCP8.5, RCP4.5)

Land Cover	RCP8.5			RCP4.5		
	2001-2050	2051-2100	2001-2100	2001-2050	2051-2100	2001-2100
Croplands	612.3	412.6	1,024.9	555.5	309.2	864.7
Pastures	0.0	0.0	0.0	0.0	0.0	0.0
Young Forests						
Boreal	0.0	0.0	0.0	0.0	0.0	0.0
Temperate	0.0	0.0	0.0	0.0	0.0	0.0
All	0.0	0.0	0.0	0.0	0.0	0.0
Old Forests						
Boreal	0.0	0.0	0.0	0.0	0.0	0.0
Temperate	0.0	0.0	0.0	0.0	0.0	0.0
All	0.0	0.0	0.0	0.0	0.0	0.0
Forests						
Boreal	0.0	0.0	0.0	0.0	0.0	0.0
Temperate	0.0	0.0	0.0	0.0	0.0	0.0
All	0.0	0.0	0.0	0.0	0.0	0.0
Temperate Grasslands	0.0	0.0	0.0	0.0	0.0	0.0
Temperate Shrublands	0.0	0.0	0.0	0.0	0.0	0.0
Tundra	0.0	0.0	0.0	0.0	0.0	0.0
Wetlands	0.0	0.0	0.0	0.0	0.0	0.0
Deserts	0.0	0.0	0.0	0.0	0.0	0.0
Total	612.3	412.6	1,024.9	555.5	309.2	864.7

Supplementary Table 13. Comparison of cumulative net nitrogen mineralization (NetNMin, Tg N) among land cover types in Northern Eurasia over different time periods under two climate change projections (RCP8.5, RCP4.5) assuming a constant seasonal active layer depth after 2000

Land Cover	RCP8.5			RCP4.5		
	2001-2050	2051-2100	2001-2100	2001-2050	2051-2100	2001-2100
Croplands	218.1	493.2	711.3	132.7	290.2	422.9
Pastures	542.4	666.9	1,209.3	500.8	547.8	1,048.6
Young Forests						
Boreal	666.0	1,065.1	1,731.1	854.7	1,042.0	1,896.7
Temperate	147.6	171.0	318.6	236.5	263.2	499.7
All	813.6	715.5	2,049.7	1,091.2	1,305.2	2,396.4
Old Forests						
Boreal	1,046.1	1,110.6	2,156.7	1,093.4	1,045.7	2,139.1
Temperate	288.1	301.9	590.0	290.5	257.7	548.2
All	1,334.2	1,412.5	2,746.7	1,383.9	1,303.4	2,687.3
Forests						
Boreal	1,712.1	2,175.7	3,887.8	1,948.1	2,087.7	4,035.8
Temperate	435.7	472.9	908.6	527.0	520.9	1,047.9
All	2,147.8	2,648.6	4,796.4	2,475.1	2,608.6	5,083.7
Temperate Grasslands	142.2	165.7	307.9	194.0	299.1	493.1
Temperate Shrublands	98.8	102.3	201.1	151.0	189.4	340.4
Tundra	145.3	163.7	309.0	143.8	155.0	298.8
Wetlands	281.2	336.2	617.4	295.1	293.6	588.7
Deserts	23.6	33.0	56.6	25.1	24.8	49.9
Total	3,599.4	4,609.6	8,209.0	3,917.6	4,408.5	8,326.1

Supplementary Table 14. Comparison of cumulative net nitrogen mineralization (NetNMin, Tg N) among land cover types on areas covered by croplands during 2000 over different time periods under two climate change projections (RCP8.5, RCP4.5) when croplands are fertilized from 1950 to 2100 (Fertilized Crop)

Land Cover	RCP8.5			RCP4.5		
	2001-2050	2051-2100	2001-2100	2001-2050	2051-2100	2001-2100
Croplands	219.9	503.9	723.8	144.9	291.7	436.6
Pastures	9.7	21.7	31.4	8.1	20.3	28.4
Young Forests						
Boreal	10.2	8.7	18.9	188.8	121.4	310.2
Temperate	21.5	17.2	38.7	116.3	113.8	230.1
All	31.7	25.9	57.6	305.1	235.2	540.3
Old Forests						
Boreal	0.0	0.0	0.0	0.0	0.0	0.0
Temperate	0.0	0.0	0.0	0.0	0.0	0.0
All	0.0	0.0	0.0	0.0	0.0	0.0
Forests						
Boreal	10.2	8.7	18.9	188.8	121.4	310.2
Temperate	21.5	17.2	38.7	116.3	113.8	230.1
All	31.7	25.9	57.6	305.1	235.2	540.3
Temperate Grasslands	2.7	4.1	6.8	35.0	103.7	138.7
Temperate Shrublands	0.1	0.2	0.3	2.3	7.6	9.9
Tundra	-0.1	0.1	0.0	0.2	0.7	0.9
Wetlands	0.8	0.7	1.5	37.6	2.1	39.7
Deserts	0.0	0.0	0.0	-0.1	0.2	0.1
Total	264.8	556.6	821.4	533.1	661.5	1,194.6

Supplementary Table 15. Comparison of legacy N fertilizer effects on cumulative net nitrogen mineralization (NetNMin, Tg N) among land cover types on areas covered by croplands during 2000 over different time periods under the RCP 8.5 climate change projection for unfertilized croplands (Unfertilized Crops) and croplands fertilized from 1950 to 2000 (Pre-2001 Fertilized Crops)

Land Cover	Unfertilized Crops			Pre-2001 Fertilized Crops		
	2001-2050	2051-2100	2001-2100	2001-2050	2051-2100	2001-2100
Croplands	311.2	326.6	637.8	631.8	468.9	1,100.7
Pastures	3.6	10.1	13.7	8.1	15.8	23.9
Young Forests						
Boreal	5.1	7.6	12.7	10.2	7.9	18.1
Temperate	9.4	10.0	19.4	18.9	12.3	31.2
All	14.5	17.6	32.1	29.1	20.2	49.3
Old Forests						
Boreal	0.0	0.0	0.0	0.0	0.0	0.0
Temperate	0.0	0.0	0.0	0.0	0.0	0.0
All	0.0	0.0	0.0	0.0	0.0	0.0
Forests						
Boreal	5.1	7.6	12.7	10.2	7.9	18.1
Temperate	9.4	10.0	19.4	18.9	12.3	31.2
All	14.5	17.6	32.1	29.1	20.2	49.3
Temperate Grasslands	1.9	3.2	5.1	2.7	3.8	6.5
Temperate Shrublands	0.1	0.2	0.3	0.1	0.2	0.3
Tundra	-0.1	-0.1	-0.2	-0.2	0.2	0.0
Wetlands	0.2	0.1	0.3	0.7	0.3	1.0
Deserts	0.0	0.0	0.0	0.0	0.0	0.0
Total	331.4	357.7	689.1	672.3	509.4	1,181.7

Supplementary Table 16. Comparison of legacy N fertilizer effects on cumulative net nitrogen mineralization (NetNMin, Tg N) among land cover types on areas covered by croplands during 2000 over different time periods under the RCP 4.5 climate change projection for unfertilized croplands (Unfertilized Crops) and croplands fertilized from 1950 to 2000 (Pre-2001 Fertilized Crops)

Land Cover	Unfertilized Crops			Pre-2001 Fertilized Crops		
	2001-2050	2051-2100	2001-2100	2001-2050	2051-2100	2001-2100
Croplands	270.3	205.4	475.7	541.8	282.9	824.7
Pastures	1.4	4.3	5.7	4.8	7.7	12.5
Young Forests						
Boreal						
Temperate	104.6	116.2	220.8	143.1	115.6	258.7
All	41.5	55.6	97.1	74.1	62.4	136.5
	146.1	171.8	317.9	217.2	178.0	395.2
Old Forests						
Boreal	0.0	0.0	0.0	0.0	0.0	0.0
Temperate	0.0	0.0	0.0	0.0	0.0	0.0
All	0.0	0.0	0.0	0.0	0.0	0.0
Forests						
Boreal	104.6	116.2	220.8	143.1	115.6	258.7
Temperate	41.5	55.6	97.1	74.1	62.4	136.5
All	146.1	171.8	317.9	217.2	178.0	395.2
Temperate Grasslands	25.4	71.0	96.4	33.8	88.7	122.5
Temperate Shrublands	3.1	6.7	9.8	3.7	7.0	10.7
Tundra	0.1	-0.4	-0.3	0.5	0.5	1.0
Wetlands	14.1	-6.2	7.9	25.7	-6.8	18.9
Deserts	-0.1	-0.2	-0.3	0.0	0.0	0.0
Total	460.4	452.4	912.8	827.5	558.0	1,385.5

Supplementary Table 17. Comparison of cumulative net nitrogen mineralization (NetNMin, Tg N) among land cover types on areas covered by pastures during 2000 over different time periods under two climate change projections (RCP8.5, RCP4.5)

Land Cover	RCP8.5			RCP4.5		
	2001-2050	2051-2100	2001-2100	2001-2050	2051-2100	2001-2100
Croplands	0.5	-0.9	-0.4	-8.4	0.0	-8.4
Pastures	535.8	647.4	1,183.2	495.8	529.9	1,025.7
Young Forests						
Boreal	5.1	8.6	13.7	77.9	104.2	182.1
Temperate	6.9	20.4	27.3	24.9	28.2	53.1
All	12.0	29.0	41.0	102.8	132.4	235.2
Old Forests						
Boreal	0.0	0.0	0.0	0.0	0.0	0.0
Temperate	0.0	0.0	0.0	0.0	0.0	0.0
All	0.0	0.0	0.0	0.0	0.0	0.0
Forests						
Boreal	5.1	8.6	13.7	77.9	104.2	182.1
Temperate	6.9	20.4	27.3	24.9	28.2	53.1
All	12.0	29.0	41.0	102.8	132.4	235.2
Temperate Grasslands	4.2	15.6	19.8	20.1	46.6	66.7
Temperate Shrublands	2.2	1.8	4.0	53.7	83.5	137.2
Tundra	-0.2	-0.6	-0.8	-1.0	-2.4	-3.4
Wetlands	0.4	-0.4	0.0	5.9	-5.5	0.4
Deserts	-0.3	-0.4	-0.7	-0.9	-1.2	-2.1
Total	554.6	691.5	1,246.1	668.0	783.3	1,451.3

Supplementary Table 18. Comparison of cumulative net nitrogen mineralization (NetNMin, Tg N) among land cover types in Northern Eurasia over different time periods during the 21st century under the RCP8.5 and RCP4.5 climate change projections assuming atmospheric N deposition remains constant at 1850 levels

Land Cover	RCP8.5			RCP4.5		
	2001-2050	2051-2100	2001-2100	2001-2050	2051-2100	2001-2100
Croplands	227.6	513.5	741.1	138.1	296.5	434.6
Pastures	596.5	670.0	1,266.5	533.7	527.4	1,061.1
Young Forests						
Boreal	685.2	1,131.6	1,816.8	860.3	1,054.9	1,915.2
Temperate	142.1	158.1	300.2	228.6	256.6	485.2
All	827.3	1,289.7	2,117.0	1,088.9	1,311.5	2,400.4
Old Forests						
Boreal	1,048.2	1,188.0	2,236.2	1,096.0	1,124.8	2,220.8
Temperate	255.2	268.1	523.3	260.3	229.2	489.5
All	1,303.4	1,456.1	2,759.5	1,356.3	1,354.0	2,710.3
Forests						
Boreal	1,733.4	2,319.6	4,053.0	1,956.3	2,179.6	4,135.9
Temperate	397.3	426.2	823.5	488.9	485.8	974.7
All	2,130.7	2,745.8	4,876.5	2,445.2	2,665.4	5,110.6
Temperate Grasslands	146.5	163.3	309.8	195.1	287.8	482.9
Temperate Shrublands	110.6	115.4	226.0	164.0	202.7	366.7
Tundra	152.3	174.0	326.3	151.7	169.6	321.3
Wetlands	274.8	345.2	620.0	290.9	295.6	586.5
Deserts	22.8	28.8	51.6	24.8	23.2	48.0
Total	3,661.8	4,756.0	8,417.8	3,943.5	4,468.2	8,411.7

Supplementary Table 19. Comparison of cumulative carbon sequestration/loss in vegetation ($VegC_{NEW}$, Pg C) among land cover types in Northern Eurasia over different time periods under two climate change projections (RCP8.5, RCP4.5) assuming a constant seasonal active layer depth after 2000

Land Cover	RCP8.5			RCP4.5		
	2001-2050	2051-2100	2001-2100	2001-2050	2051-2100	2001-2100
Croplands	-0.1	0.1	0.0	-0.3	0.0	-0.3
Pastures	0.2	-0.3	-0.1	0.0	0.0	0.0
Young Forests						
Boreal	0.4	12.6	13.0	11.2	8.4	19.6
Temperate	2.5	2.5	5.0	4.6	3.8	8.4
All	2.9	15.1	18.0	15.8	12.2	28.0
Old Forests						
Boreal	9.4	14.2	23.6	8.9	6.1	15.0
Temperate	1.8	2.3	4.1	1.7	0.7	2.4
All	11.2	16.5	27.7	10.6	6.8	17.4
Forests						
Boreal	9.8	26.8	36.6	20.1	14.5	34.6
Temperate	4.3	4.8	9.1	6.3	4.6	10.8
All	14.1	31.6	45.7	26.4	19.0	45.4
Temperate Grasslands	0.1	0.1	0.2	0.5	0.1	0.6
Temperate Shrublands	0.2	0.1	0.3	0.4	0.1	0.5
Tundra	0.7	0.8	1.5	0.6	0.2	0.8
Wetlands	1.6	2.9	4.5	2.2	1.1	3.3
Deserts	0.3	0.2	0.5	0.2	0.0	0.2
Total	17.1	35.5	52.6	30.0	20.5	50.5

Supplementary Table 20. Comparison of cumulative carbon sequestration in vegetation ($VegC_{NEW}$, Pg C) among land cover types on areas covered by croplands during 2000 over different time periods under two climate change projections (RCP8.5, RCP4.5) when croplands are fertilized from 1950 to 2100 (Fertilized Crop)

Land Cover	RCP8.5			RCP4.5		
	2001-2050	2051-2100	2001-2100	2001-2050	2051-2100	2001-2100
Croplands	0.1	-0.1	0.0	-0.1	0.2	0.1
Pastures	-0.1	0.2	0.1	-0.1	0.1	0.0
Young Forests						
Boreal	0.3	0.3	0.6	4.6	3.8	8.4
Temperate	0.5	0.2	0.7	2.0	2.6	4.6
All	0.8	0.5	1.3	6.6	6.4	13.0
Old Forests						
Boreal	0.0	0.0	0.0	0.0	0.0	0.0
Temperate	0.0	0.0	0.0	0.0	0.0	0.0
All	0.0	0.0	0.0	0.0	0.0	0.0
Forests						
Boreal	0.3	0.3	0.6	4.6	3.8	8.4
Temperate	0.5	0.	0.7	2.0	2.6	4.6
All	0.8	0.5	1.3	6.6	6.4	13.0
Temperate Grasslands	0.0	0.0	0.0	0.3	0.1	0.4
Temperate Shrublands	0.0	0.0	0.0	0.0	0.0	0.0
Tundra	0.0	0.0	0.0	0.0	0.0	0.0
Wetlands	0.0	0.1	0.1	0.4	0.4	0.8
Deserts	0.0	0.0	0.0	0.0	0.0	0.0
Total	0.8	0.7	1.5	7.1	7.2	14.3

Supplementary Table 21. Comparison of legacy N fertilizer effects on cumulative carbon sequestration/loss in vegetation ($VegC_{NEW}$, Pg C) among land cover types on areas covered by croplands during 2000 over different time periods under the RCP 8.5 climate change projection for unfertilized croplands (Unfertilized Crops) and croplands fertilized from 1950 to 2000 (Pre-2001 Fertilized Crops)

Land Cover	Unfertilized Crops			Pre-2001 Fertilized Crops		
	2001-2050	2051-2100	2001-2100	2001-2050	2051-2100	2001-2100
Croplands	0.1	-0.1	0.0	0.1	-0.1	0.0
Pastures	0.0	0.0	0.0	0.0	0.0	0.0
Young Forests						
Boreal	0.3	0.2	0.5	0.3	0.2	0.5
Temperate	0.5	0.2	0.7	0.5	0.3	0.8
All	0.8	0.4	1.2	0.8	0.5	1.3
Old Forests						
Boreal	0.0	0.0	0.0	0.0	0.0	0.0
Temperate	0.0	0.0	0.0	0.0	0.0	0.0
All	0.0	0.0	0.0	0.0	0.0	0.0
Forests						
Boreal	0.3	0.2	0.5	0.3	0.2	0.5
Temperate	0.5	0.2	0.7	0.5	0.3	0.8
All	0.8	0.4	1.2	0.8	0.5	1.3
Temperate Grasslands	0.1	0.0	0.1	0.0	0.0	0.0
Temperate Shrublands	0.0	0.0	0.0	0.0	0.0	0.0
Tundra	0.0	0.0	0.0	0.0	0.0	0.0
Wetlands	0.0	0.1	0.1	0.0	0.0	0.0
Deserts	0.0	0.0	0.0	0.0	0.0	0.0
Total	1.0	0.4	1.4	0.9	0.4	1.3

Supplementary Table 22. Comparison of legacy N fertilizer effects on cumulative carbon sequestration/loss in vegetation ($VegC_{NEW}$, Pg C) among land cover types on areas covered by croplands during 2000 over different time periods under the RCP 4.5 climate change projection for unfertilized croplands (Unfertilized Crops) and croplands fertilized from 1950 to 2000 (Pre-2001 Fertilized Crops)

Land Cover	Unfertilized Crops			Pre-2001 Fertilized Crops		
	2001-2050	2051-2100	2001-2100	2001-2050	2051-2100	2001-2100
Croplands	0.0	0.0	0.0	-0.1	0.1	0.0
Pastures	-0.1	0.1	0.0	0.0	0.0	0.0
Young Forests						
Boreal	4.7	3.0	7.7	4.8	3.3	8.1
Temperate	2.0	1.9	3.9	1.9	2.5	4.4
All	6.7	4.9	11.6	6.7	5.8	12.5
Old Forests						
Boreal	0.0	0.0	0.0	0.0	0.0	0.0
Temperate	0.0	0.0	0.0	0.0	0.0	0.0
All	0.0	0.0	0.0	0.0	0.0	0.0
Forests						
Boreal	4.7	3.0	7.7	4.8	3.3	8.1
Temperate	2.0	1.9	3.9	1.9	2.5	4.4
All	6.7	4.9	11.6	6.7	5.8	12.5
Temperate Grasslands	0.2	0.1	0.3	0.3	0.1	0.4
Temperate Shrublands	0.0	0.0	0.0	0.0	0.1	0.1
Tundra	0.1	-0.1	0.0	0.0	0.0	0.0
Wetlands	0.5	0.3	0.8	0.4	0.3	0.7
Deserts	0.0	0.0	0.0	0.0	0.0	0.0
Total	7.4	5.3	12.7	7.3	6.4	13.7

Supplementary Table 23. Comparison of cumulative carbon sequestration in vegetation ($VegC_{NEW}$, Pg C) among land cover types on areas covered by pastures during 2000 over different time periods under two climate change projections (RCP8.5, RCP4.5)

Land Cover	RCP8.5			RCP4.5		
	2001-2050	2051-2100	2001-2100	2001-2050	2051-2100	2001-2100
Croplands	0.1	-0.1	0.0	0.0	0.0	0.0
Pastures	0.2	0.2	0.4	0.2	0.0	0.2
Young Forests						
Boreal	0.3	0.3	0.6	4.8	2.0	6.8
Temperate	0.3	0.9	1.2	1.3	0.4	1.7
All	0.6	1.2	1.8	6.1	2.4	8.5
Old Forests						
Boreal	0.0	0.0	0.0	0.0	0.0	0.0
Temperate	0.0	0.0	0.0	0.0	0.0	0.0
All	0.0	0.0	0.0	0.0	0.0	0.0
Forests						
Boreal	0.3	0.3	0.6	4.8	2.0	6.8
Temperate	0.3	0.9	1.2	1.3	0.4	1.7
All	0.6	1.2	1.8	6.1	2.4	8.5
Temperate Grasslands	0.1	0.0	0.1	0.1	0.1	0.2
Temperate Shrublands	0.0	0.0	0.0	0.2	0.1	0.3
Tundra	0.0	0.0	0.0	0.1	0.0	0.1
Wetlands	0.0	0.0	0.0	0.3	0.1	0.4
Deserts	0.0	0.0	0.0	0.0	0.0	0.0
Total	1.0	1.3	2.3	7.0	2.7	9.7

Supplementary Table 24. Comparison of cumulative carbon sequestration/loss in vegetation ($VegC_{NEW}$, Pg C) among land cover types in Northern Eurasia over different time periods during the 21st century under the RCP8.5 and RCP4.5 climate change projections assuming atmospheric N deposition remains constant at 1850 levels

Land Cover	RCP8.5			RCP4.5		
	2001-2050	2051-2100	2001-2100	2001-2050	2051-2100	2001-2100
Croplands	0.1	0.0	0.1	-0.2	0.0	-0.2
Pastures	0.1	-0.1	0.0	0.0	0.0	0.0
Young Forests						
Boreal	1.1	14.1	15.2	11.4	8.2	19.6
Temperate	2.2	1.7	3.9	4.4	3.5	7.9
All	3.3	15.8	19.1	15.8	11.7	27.5
Old Forests						
Boreal	9.2	17.0	26.2	9.0	8.4	17.4
Temperate	1.1	1.4	2.5	1.1	0.3	1.4
All	10.3	18.4	28.7	10.1	8.7	18.8
Forests						
Boreal	10.3	31.1	41.4	20.4	16.6	37.0
Temperate	3.3	3.1	6.4	5.5	3.8	9.3
All	13.6	34.2	47.8	25.9	20.4	46.3
Temperate Grasslands	0.2	-0.1	0.1	0.5	0.0	0.5
Temperate Shrublands	0.1	0.1	0.2	0.4	0.0	0.4
Tundra	0.7	1.1	1.8	0.5	0.5	1.0
Wetlands	1.1	3.0	4.1	1.9	1.1	3.0
Deserts	0.2	0.4	0.6	0.1	0.1	0.2
Total	16.1	38.6	54.7	29.1	22.1	51.2

Supplementary Table 25. Fate of area (million ha) covered by croplands in 2000 to various land covers/land uses in Northern Eurasia over the 21st century under the RCP8.5 and RCP4.5 climate change projections and land-use transitions described by Hurtt et al.[11]

Land Cover	2000	RCP8.5		RCP4.5	
		2050	2100	2050	2100
Croplands	289	275	270	175	148
Pastures	0	6	9	5	9
Young Forests					
Boreal	0	2	2	34	35
Temperate	0	4	6	26	37
All	0	6	8	60	72
Old Forests					
Boreal	0	0	0	0	0
Temperate	0	0	0	0	0
All	0	0	0	0	0
All Forests					
Boreal	0	2	2	34	35
Temperate	0	4	6	26	37
All	0	6	8	60	72
Temperate Grasslands	0	2	2	41	50
Temperate Shrublands	0	0	0	4	5
Tundra	0	0	0	1	1
Wetlands	0	0	0	3	4
Deserts	0	0	0	0	0
Lakes	0	0	0	0	0
Glaciers	0	0	0	0	0
Total	289	289	289	289	289

Supplementary Table 26. Fate on area (million ha) covered by pastures in 2000 to various land covers/land uses in Northern Eurasia over the 21st century under the RCP8.5 and RCP4.5 climate change projections and land-use transitions described by Hurtt et al.[11]

Land Cover	2000	RCP8.5		RCP4.5	
		2050	2100	2050	2100
Croplands	0	1	3	8	7
Pastures	618	593	592	479	478
Young Forests					
Boreal	0	2	3	30	31
Temperate	0	7	8	17	16
All	0	9	11	47	47
Old Forests					
Boreal	0	0	0	0	0
Temperate	0	0	0	0	0
All	0	0	0	0	0
All Forests					
Boreal	0	2	3	30	31
Temperate	0	7	8	17	16
All	0	9	11	47	47
Temperate Grasslands	0	13	11	36	37
Temperate Shrublands	0	2	1	44	45
Tundra	0	0	0	1	1
Wetlands	0	0	0	2	2
Deserts	0	0	0	1	1
Lakes	0	0	0	0	0
Glaciers	0	0	0	0	0
Total	618	618	618	618	618

Supplementary Table 27. Comparison of cumulative carbon sequestration/loss in soil organic matter ($TotSOC_{NEW}$, Pg C) among land cover types in Northern Eurasia over different time periods under two climate change projections (RCP8.5, RCP4.5) assuming a constant seasonal active layer depth after 2000

Land Cover	RCP8.5			RCP4.5		
	2001-2050	2051-2100	2001-2100	2001-2050	2051-2100	2001-2100
Croplands	-1.6	-1.7	-3.3	0.2	-0.6	-0.4
Pastures	-1.4	-0.9	-2.3	-0.4	-0.4	-0.8
Young Forests						
Boreal	-2.4	-1.8	-4.2	-1.4	0.7	-0.7
Temperate	0.4	1.1	1.5	0.1	2.8	2.9
All	-2.0	-0.7	-2.7	-1.3	3.5	2.2
Old Forests						
Boreal	1.8	2.8	4.6	2.4	6.9	9.3
Temperate	0.6	-0.1	0.5	0.7	0.1	0.8
All	2.4	2.7	5.1	3.1	7.0	10.1
Forests						
Boreal	-0.6	1.0	0.4	1.0	7.5	8.5
Temperate	1.0	1.0	2.0	0.8	3.0	3.8
All	0.4	2.0	2.4	1.8	10.5	12.3
Temperate Grasslands	0.1	0.0	0.1	-0.1	0.0	-0.1
Temperate Shrublands	0.5	0.6	1.1	0.1	0.2	0.3
Tundra	1.1	5.8	6.9	0.9	4.0	4.9
Wetlands	-0.3	0.0	-0.3	-0.2	0.5	0.3
Deserts	0.8	2.1	2.9	1.0	1.2	2.2
Total	-0.4	7.9	7.5	3.3	15.4	18.7

Supplementary Table 28. Comparison of cumulative carbon sequestration/loss in soil organic matter ($TotSOC_{NEW}$, Pg C) among land cover types on areas covered by croplands during 2000 over different time periods under two climate change projections (RCP8.5, RCP4.5) when croplands are fertilized from 1950 to 2100 (Fertilized Crop)

Land Cover	RCP8.5			RCP4.5		
	2001-2050	2051-2100	2001-2100	2001-2050	2051-2100	2001-2100
Croplands	-1.9	-1.3	-3.2	0.0	-0.7	-0.7
Pastures	-0.1	-0.1	-0.2	-0.1	-0.2	-0.3
Young Forests						
Boreal	0.0	-0.1	-0.1	-1.4	0.7	-0.7
Temperate	-0.1	0.3	0.2	-0.7	0.9	0.2
All	-0.1	0.2	0.1	-2.1	1.6	-0.5
Old Forests						
Boreal	0.0	0.0	0.0	0.0	0.0	0.0
Temperate	0.0	0.0	0.0	0.0	0.0	0.0
All	0.0	0.0	0.0	0.0	0.0	0.0
Forests						
Boreal	0.0	-0.1	-0.1	-1.4	0.7	-0.7
Temperate	-0.1	0.3	0.2	-0.7	0.9	0.2
All	-0.1	0.2	0.1	-2.1	1.6	-0.5
Temperate Grasslands	0.0	-0.1	-0.1	-0.2	0.0	-0.2
Temperate Shrublands	0.0	0.0	0.0	0.0	-0.1	-0.1
Tundra	0.0	0.0	0.0	-0.1	0.1	0.0
Wetlands	0.0	0.0	0.0	-0.2	0.1	-0.1
Deserts	0.0	-0.1	-0.1	0.0	0.0	0.0
Total	-2.1	-1.4	-3.5	-2.7	0.8	-1.9

Supplementary Table 29. Comparison of legacy N fertilizer effects on cumulative carbon sequestration/loss in soil matter carbon ($TotSOC_{NEW}$, Pg C) among land cover types on areas covered by croplands during 2000 over different time periods under the RCP 8.5 climate change projection for unfertilized croplands (Unfertilized Crops) and croplands fertilized from 1950 to 2000 (Pre-2001 Fertilized Crops)

Land Cover	Unfertilized Crops			Pre-2001 Fertilized Crops		
	2001-2050	2051-2100	2001-2100	2001-2050	2051-2100	2001-2100
Croplands	-1.2	-0.3	-1.5	-7.5	-0.7	-8.2
Pastures	0.0	0.0	0.0	-0.1	0.0	-0.1
Young Forests						
Boreal	0.0	0.1	0.1	0.0	0.0	0.0
Temperate	0.1	0.1	0.2	0.0	0.1	0.1
All	0.1	0.2	0.3	0.0	0.1	0.1
Old Forests						
Boreal	0.0	0.0	0.0	0.0	0.0	0.0
Temperate	0.0	0.0	0.0	0.0	0.0	0.0
All	0.0	0.0	0.0	0.0	0.0	0.0
Forests						
Boreal	0.0	0.1	0.1	0.0	0.0	0.0
Temperate	0.1	0.1	0.2	0.0	0.1	0.1
All	0.1	0.2	0.3	0.0	0.1	0.1
Temperate Grasslands	0.0	-0.1	-0.1	0.0	0.0	0.0
Temperate Shrublands	0.0	0.0	0.0	0.0	0.0	0.0
Tundra	0.0	0.0	0.0	0.0	0.0	0.0
Wetlands	0.0	0.0	0.0	0.0	0.0	0.0
Deserts	0.0	0.0	0.0	0.0	0.0	0.0
Total	-1.1	-0.2	-1.3	-7.6	-0.6	-8.2

Supplementary Table 30. Comparison of legacy N fertilizer effects on cumulative carbon sequestration/loss in soil organic matter ($TotSOC_{NEW}$, Pg C) among land cover types on areas covered by croplands during 2000 over different time periods under the RCP 4.5 climate change projection for unfertilized croplands (Unfertilized Crops) and croplands fertilized from 1950 to 2000 (Pre-2001 Fertilized Crops)

Land Cover	Unfertilized Crops			Pre-2001 Fertilized Crops		
	2001-2050	2051-2100	2001-2100	2001-2050	2051-2100	2001-2100
Croplands	-0.9	-0.2	-1.1	-6.5	-0.5	-7.0
Pastures	0.0	-0.1	-0.1	-0.2	0.0	-0.2
Young Forests						
Boreal	0.2	0.8	1.0	-0.3	1.0	0.7
Temperate	0.1	1.2	1.3	0.1	1.0	1.1
All	0.3	2.0	2.3	-0.2	2.0	1.8
Old Forests						
Boreal	0.0	0.0	0.0	0.0	0.0	0.0
Temperate	0.0	0.0	0.0	0.0	0.0	0.0
All	0.0	0.0	0.0	0.0	0.0	0.0
Forests						
Boreal	0.2	0.8	1.0	-0.3	1.0	0.7
Temperate	0.1	1.2	1.3	0.1	1.0	1.1
All	0.3	2.0	2.3	-0.2	2.0	1.8
Temperate Grasslands	0.1	0.0	0.1	0.1	-0.1	0.0
Temperate Shrublands	0.0	0.0	0.0	0.0	0.0	0.0
Tundra	0.0	0.1	0.1	0.0	0.1	0.1
Wetlands	0.0	0.1	0.1	0.0	0.1	0.1
Deserts	0.0	0.0	0.0	0.0	0.0	0.0
Total	-0.5	1.9	1.4	-6.8	1.6	-5.2

Supplementary Table 31. Comparison of cumulative carbon sequestration/loss in soil organic matter ($TotSOC_{NEW}$, Pg C) among land cover on areas covered by pastures during 2000 over different time periods under two climate change projections (RCP8.5, RCP4.5)

Land Cover	RCP8.5			RCP4.5		
	2001-2050	2051-2100	2001-2100	2001-2050	2051-2100	2001-2100
Croplands	0.0	0.1	0.1	0.2	0.1	0.3
Pastures	-1.0	-0.9	-1.9	-0.4	-0.3	-0.7
Young Forests						
Boreal	0.0	0.0	0.0	0.3	0.8	1.1
Temperate	0.1	0.3	0.4	0.2	0.5	0.7
All	0.1	0.3	0.4	0.5	1.3	1.8
Old Forests						
Boreal	0.0	0.0	0.0	0.0	0.0	0.0
Temperate	0.0	0.0	0.0	0.0	0.0	0.0
All	0.0	0.0	0.0	0.0	0.0	0.0
Forests						
Boreal	0.0	0.0	0.0	0.3	0.8	1.1
Temperate	0.1	0.3	0.4	0.2	0.5	0.7
All	0.1	0.3	0.4	0.5	1.3	1.8
Temperate Grasslands	0.0	0.1	0.1	0.1	-0.1	0.0
Temperate Shrublands	0.0	0.0	0.0	-0.4	-0.2	-0.6
Tundra	0.0	0.0	0.0	0.0	0.0	0.0
Wetlands	0.0	0.0	0.0	0.1	0.0	0.1
Deserts	0.0	0.0	0.0	0.0	0.0	0.0
Total	-0.9	-0.4	-1.3	0.1	0.8	0.9

Supplementary Table 32. Comparison of cumulative carbon sequestration/loss in soil organic matter ($TotSOC_{NEW}$, Pg C) among land cover types in Northern Eurasia over different time periods during the 21st century under the RCP8.5 and RCP4.5 climate change projections assuming atmospheric N deposition remains constant at 1850 levels.

Land Cover	RCP8.5			RCP4.5		
	2001-2050	2051-2100	2001-2100	2001-2050	2051-2100	2001-2100
Croplands	-1.8	-1.5	-3.3	0.1	-0.4	-0.3
Pastures	-1.7	-1.2	-2.9	-0.7	-0.6	-1.3
Young Forests						
Boreal	-4.1	-1.6	-5.7	-2.4	0.8	-1.6
Temperate	0.1	1.1	1.2	-0.1	2.2	2.1
All	-4.0	-0.5	-4.5	-2.5	3.0	0.5
Old Forests						
Boreal	-4.8	-2.0	-6.8	-4.3	2.8	-1.5
Temperate	-0.5	0.1	-0.4	-0.4	0.2	-0.2
All	-5.3	-1.9	-7.2	-4.7	3.0	-1.7
Forests						
Boreal	-8.9	-3.6	-12.5	-6.7	3.6	-3.1
Temperate	-0.4	1.2	0.8	-0.5	2.4	1.9
All	-9.3	-2.4	-11.7	-7.2	6.0	-1.2
Temperate Grasslands	-0.2	0.1	-0.1	-0.3	0.0	-0.3
Temperate Shrublands	0.3	0.5	0.8	-0.2	0.1	-0.1
Tundra	-0.6	-4.1	-4.7	-0.3	-0.6	-0.9
Wetlands	-0.6	-1.0	-1.6	-0.5	-0.2	-0.7
Deserts	0.6	0.8	1.4	0.7	0.7	1.4
Total	-13.3	-8.8	-22.1	-8.4	5.0	-3.4

Supplementary Table 33. Comparison of cumulative net ecosystem carbon balance (NECB, Pg C) among land cover types in Northern Eurasia over different time periods under two climate change projections (RCP8.5, RCP4.5) assuming a constant seasonal active layer depth after 2000

Land Cover	RCP8.5			RCP4.5		
	2001-2050	2051-2100	2001-2100	2001-2050	2051-2100	2001-2100
Croplands	-2.0	-1.5	-3.5	-0.5	-0.5	-1.0
Pastures	-1.5	-1.3	-2.8	-0.4	-0.7	-1.1
Young Forests						
Boreal	-0.9	11.1	10.2	10.3	9.5	19.8
Temperate	3.0	3.4	6.4	5.0	6.4	11.4
All	2.1	14.5	16.6	15.3	15.9	31.2
Old Forests						
Boreal	11.1	16.4	27.5	11.4	12.8	24.2
Temperate	2.5	2.9	5.4	2.3	1.1	3.4
All	13.6	19.3	32.9	13.7	13.9	27.6
Forests						
Boreal	10.2	27.5	37.7	21.7	22.3	44.0
Temperate	5.5	6.3	11.8	7.3	7.5	14.8
All	15.7	33.8	49.5	29.0	29.8	58.8
Temperate Grasslands	0.2	0.1	0.3	0.4	0.1	0.5
Temperate Shrublands	0.7	0.7	1.4	0.5	0.3	0.8
Tundra	1.8	6.6	8.4	1.4	4.2	5.6
Wetlands	1.4	3.0	4.4	2.0	1.7	3.7
Deserts	1.1	2.3	3.4	1.2	1.3	2.5
Total	17.4	43.7	61.1	33.6	36.2	69.8

Supplementary Table 34. Comparison of cumulative net ecosystem carbon balance (NECB, Pg C) among land cover types on areas covered by croplands during 2000 over different time periods under two climate change projections (RCP8.5, RCP4.5) when croplands are fertilized from 1950 to 2100 (Fertilized Crop)

Land Cover	RCP8.5			RCP4.5		
	2001-2050	2051-2100	2001-2100	2001-2050	2051-2100	2001-2100
Croplands	-2.0	-1.4	-3.4	-0.3	-0.5	-0.8
Pastures	-0.1	-0.1	-0.2	-0.2	-0.1	-0.3
Young Forests						
Boreal	0.3	0.2	0.5	3.1	4.6	7.7
Temperate	0.4	0.4	0.8	1.3	3.5	4.8
All	0.7	0.6	1.3	4.4	8.1	12.5
Old Forests						
Boreal	0.0	0.0	0.0	0.0	0.0	0.0
Temperate	0.0	0.0	0.0	0.0	0.0	0.0
All	0.0	0.0	0.0	0.0	0.0	0.0
Forests						
Boreal	0.3	0.2	0.5	3.1	4.6	7.7
Temperate	0.4	0.4	0.8	1.3	3.5	4.8
All	0.7	0.6	1.3	4.4	8.1	12.5
Temperate Grasslands	0.0	0.0	0.0	0.1	0.0	0.1
Temperate Shrublands	0.0	0.0	0.0	0.0	-0.1	-0.1
Tundra	0.0	0.0	0.0	0.0	0.0	0.0
Wetlands	0.0	0.0	0.0	0.3	0.4	0.7
Deserts	0.0	0.0	0.0	0.0	0.0	0.0
Total	-1.4	-0.9	-2.3	4.3	7.8	12.1

Supplementary Table 35. Comparison of legacy N fertilizer effects on cumulative net ecosystem carbon balance (NECB, Pg C) among land cover types on areas covered by croplands during 2000 over different time periods under the RCP 8.5 climate change projection for unfertilized croplands (Unfertilized Crops) and croplands fertilized from 1950 to 2000 (Pre-2001 Fertilized Crops)

Land Cover	Unfertilized Crops			Pre-2001 Fertilized Crops		
	2001-2050	2051-2100	2001-2100	2001-2050	2051-2100	2001-2100
Croplands	-1.4	-0.4	-1.8	-7.7	-0.8	-8.5
Pastures	0.0	0.0	0.0	-0.1	0.0	-0.1
Young Forests						
Boreal	0.3	0.2	0.5	0.3	0.2	0.5
Temperate	0.6	0.4	1.0	0.5	0.4	0.9
All	0.9	0.6	1.5	0.8	0.6	1.4
Old Forests						
Boreal	0.0	0.0	0.0	0.0	0.0	0.0
Temperate	0.0	0.0	0.0	0.0	0.0	0.0
All	0.0	0.0	0.0	0.0	0.0	0.0
Forests						
Boreal	0.3	0.2	0.5	0.3	0.2	0.5
Temperate	0.6	0.4	1.0	0.5	0.4	0.9
All	0.9	0.6	1.5	0.8	0.6	1.4
Temperate Grasslands	0.0	0.0	0.0	0.0	0.0	0.0
Temperate Shrublands	0.0	0.0	0.0	0.0	0.0	0.0
Tundra	0.0	0.0	0.0	0.0	0.0	0.0
Wetlands	0.0	0.0	0.0	0.0	0.0	0.0
Deserts	0.0	0.0	0.0	0.0	0.0	0.0
Total	-0.5	0.2	-0.3	-7.0	-0.2	-7.2

Supplementary Table 36. Comparison of legacy N fertilizer effects on cumulative net ecosystem carbon balance (NECB, Pg C) among land cover types on areas covered by croplands during 2000 over different time periods under the RCP 4.5 climate change projection for unfertilized croplands (Unfertilized Crops) and croplands fertilized from 1950 to 2000 (Pre-2001 Fertilized Crops)

Land Cover	Unfertilized Crops			Pre-2001 Fertilized Crops		
	2001-2050	2051-2100	2001-2100	2001-2050	2051-2100	2001-2100
Croplands	-1.1	-0.2	-1.3	-6.8	-0.4	-7.2
Pastures	-0.1	0.0	-0.1	-0.1	-0.1	-0.2
Young Forests						
Boreal	4.9	3.7	8.6	4.5	4.2	8.7
Temperate	2.1	3.2	5.3	1.9	3.7	5.6
All	7.0	6.9	13.9	6.4	7.9	14.3
Old Forests						
Boreal	0.0	0.0	0.0	0.0	0.0	0.0
Temperate	0.0	0.0	0.0	0.0	0.0	0.0
All	0.0	0.0	0.0	0.0	0.0	0.0
Forests						
Boreal	4.9	3.7	8.6	4.5	4.2	8.7
Temperate	2.1	3.2	5.3	1.9	3.7	5.6
All	7.0	6.9	13.9	6.4	7.9	14.3
Temperate Grasslands	0.3	0.1	0.4	0.3	0.1	0.4
Temperate Shrublands	0.0	0.0	0.0	0.0	0.0	0.0
Tundra	0.0	0.1	0.1	0.0	0.0	0.0
Wetlands	0.4	0.4	0.8	0.4	0.4	0.8
Deserts	0.0	0.0	0.0	0.0	0.0	0.0
Total	6.5	7.3	13.8	0.2	7.9	8.1

Supplementary Table 37. Comparison of cumulative net ecosystem carbon balance (NECB, Pg C) among land cover types in Northern Eurasia over different time periods during the 21st century under the RCP8.5 and RCP4.5 climate change projections assuming atmospheric N deposition remains constant at 1850 levels.

Land Cover	RCP8.5			RCP4.5		
	2001-2050	2051-2100	2001-2100	2001-2050	2051-2100	2001-2100
Croplands	-2.0	-1.5	-3.5	-0.3	-0.6	-0.9
Pastures	-1.9	-1.4	-3.3	-0.8	-0.7	-1.5
Young Forests						
Boreal	-2.1	12.9	10.8	9.5	9.4	18.9
Temperate	2.5	7.7	5.2	4.5	5.6	10.1
All	0.4	15.6	16.0	14.0	15.0	29.0
Old Forests						
Boreal	4.5	15.0	19.5	4.8	11.1	15.9
Temperate	0.5	1.5	2.0	0.6	0.5	1.1
All	5.0	16.5	21.5	5.4	11.6	17.0
Forests						
Boreal	2.4	27.9	30.3	14.3	20.5	34.8
Temperate	3.0	4.1	7.2	5.1	6.1	11.2
All	5.4	32.1	37.5	19.4	26.6	46.0
Temperate Grasslands	0.0	0.0	0.0	0.2	0.0	0.2
Temperate Shrublands	0.4	0.6	1.0	0.1	0.2	0.3
Tundra	0.1	-3.1	-3.0	0.2	-0.1	0.1
Wetlands	0.6	2.3	2.9	1.5	1.1	2.6
Deserts	0.9	1.1	2.0	0.8	0.9	1.7
Total	3.5	30.1	33.6	21.1	27.4	48.5

Supplementary Table 38. Comparison of cumulative net ecosystem carbon balance (NECB, Pg C) among land cover types on areas covered by pastures during 2000 over different time periods under two climate change projections (RCP8.5, RCP4.5)

Land Cover	RCP8.5			RCP4.5		
	2001-2050	2051-2100	2001-2100	2001-2050	2051-2100	2001-2100
Croplands	0.0	0.0	0.0	0.2	0.1	0.3
Pastures	-1.0	-0.8	-1.8	-0.2	-0.5	-0.7
Young Forests						
Boreal	0.3	0.4	0.7	5.1	2.7	7.8
Temperate	0.3	1.2	1.5	1.4	1.1	2.5
All	0.6	1.6	2.2	6.5	3.8	10.3
Old Forests						
Boreal	0.0	0.0	0.0	0.0	0.0	0.0
Temperate	0.0	0.0	0.0	0.0	0.0	0.0
All	0.0	0.0	0.0	0.0	0.0	0.0
Forests						
Boreal	0.3	0.4	0.7	5.1	2.7	7.8
Temperate	0.3	1.2	1.5	1.4	1.1	2.5
All	0.6	1.6	2.2	6.5	3.8	10.3
Temperate Grasslands	0.1	0.1	0.2	0.2	0.0	0.2
Temperate Shrublands	0.0	0.0	0.0	-0.2	-0.1	-0.3
Tundra	0.0	0.0	0.0	0.0	0.1	0.1
Wetlands	0.0	0.1	0.1	0.4	0.1	0.5
Deserts	0.0	0.0	0.0	0.0	0.0	0.0
Total	-0.3	1.0	0.7	6.9	3.5	10.4

Supplementary Table 39. Comparison of cumulative heterotrophic respiration (R_H, Pg C) among land cover types on areas covered by croplands during 2000 over different time periods under two climate change projections (RCP8.5, RCP4.5) when croplands are fertilized from 1950 to 2100 (Fertilized Crop)

Land Cover	RCP8.5			RCP4.5		
	2001-2050	2051-2100	2001-2100	2001-2050	2051-2100	2001-2100
Croplands	49.2	44.4	93.6	40.5	26.6	67.1
Pastures	0.3	0.6	0.9	0.2	0.3	0.5
Young Forests						
Boreal	0.2	0.6	0.8	3.2	7.9	11.1
Temperate	0.6	1.3	1.9	2.1	6.3	8.4
All	0.8	1.9	2.7	5.3	14.2	19.5
Old Forests						
Boreal	0.0	0.0	0.0	0.0	0.0	0.0
Temperate	0.0	0.0	0.0	0.0	0.0	0.0
All	0.0	0.0	0.0	0.0	0.0	0.0
Forests						
Boreal	0.2	0.6	0.8	3.2	7.9	11.1
Temperate	0.6	1.3	1.9	2.1	6.3	8.4
All	0.8	1.9	2.7	5.3	14.2	19.5
Temperate Grasslands	0.2	0.3	0.5	2.2	6.5	8.7
Temperate Shrublands	0.0	0.0	0.0	0.1	0.3	0.4
Tundra	0.0	0.0	0.0	0.1	0.1	0.2
Wetlands	0.0	0.0	0.0	0.3	0.7	1.0
Deserts	0.0	0.1	0.1	0.0	0.0	0.0
Total	50.5	47.3	97.8	48.7	48.7	97.4

Supplementary Table 40. Comparison of legacy N fertilizer effects on cumulative heterotrophic respiration (R_H, Pg C) among land cover types on areas covered by croplands during 2000 over different time periods under the RCP 8.5 climate change projection for unfertilized croplands (Unfertilized Crops) and croplands fertilized from 1950 to 2000 (Pre-2001 Fertilized Crops)

Land Cover	Unfertilized Crops			Pre-2001 Fertilized Crops		
	2001-2050	2051-2100	2001-2100	2001-2050	2051-2100	2001-2100
Croplands	15.2	16.3	31.5	23.0	18.4	41.4
Pastures	0.2	0.5	0.7	0.3	0.5	0.8
Young Forests						
Boreal	0.2	0.4	0.6	0.2	0.5	0.7
Temperate	0.4	1.3	1.7	0.5	1.4	1.9
All	0.6	1.7	2.3	0.7	1.9	2.6
Old Forests						
Boreal	0.0	0.0	0.0	0.0	0.0	0.0
Temperate	0.0	0.0	0.0	0.0	0.0	0.0
All	0.0	0.0	0.0	0.0	0.0	0.0
Forests						
Boreal	0.2	0.4	0.6	0.2	0.5	0.7
Temperate	0.4	1.3	1.7	0.5	1.4	1.9
All	0.6	1.7	2.3	0.7	1.9	2.6
Temperate Grasslands	0.1	0.3	0.4	0.2	0.2	0.4
Temperate Shrublands	0.0	0.0	0.0	0.0	0.0	0.0
Tundra	0.0	0.0	0.0	0.0	0.0	0.0
Wetlands	0.0	0.0	0.0	0.0	0.1	0.1
Deserts	0.0	0.0	0.0	0.0	0.0	0.0
Total	16.1	18.8	34.9	24.2	21.1	45.3

Supplementary Table 41. Comparison of legacy N fertilizer effects on cumulative heterotrophic respiration (R_H, Pg C) among land cover types on areas covered by croplands during 2000 over different time periods under the RCP 4.5 climate change projection for unfertilized croplands (Unfertilized Crops) and croplands fertilized from 1950 to 2000 (Pre-2001 Fertilized Crops)

Land Cover	Unfertilized Crops			Pre-2001 Fertilized Crops		
	2001-2050	2051-2100	2001-2100	2001-2050	2051-2100	2001-2100
Croplands	13.3	9.9	23.2	20.5	11.3	31.8
Pastures	0.1	0.2	0.3	0.2	0.2	0.4
Young Forests						
Boreal	1.8	7.5	9.3	2.2	7.7	9.9
Temperate	1.2	5.2	6.4	1.4	5.8	7.2
All	3.0	12.7	15.7	3.6	13.5	17.1
Old Forests						
Boreal	0.0	0.0	0.0	0.0	0.0	0.0
Temperate	0.0	0.0	0.0	0.0	0.0	0.0
All	0.0	0.0	0.0	0.0	0.0	0.0
Forests						
Boreal	1.8	7.5	9.3	2.2	7.7	9.9
Temperate	1.2	5.2	6.4	1.4	5.8	7.2
All	3.0	12.7	15.7	3.6	13.5	17.1
Temperate Grasslands	1.5	4.7	6.2	1.9	5.8	7.7
Temperate Shrublands	0.1	0.2	0.3	0.1	0.2	0.3
Tundra	0.0	0.1	0.1	0.0	0.1	0.1
Wetlands	0.1	0.7	0.8	0.2	0.7	0.9
Deserts	0.0	0.0	0.0	0.0	0.0	0.0
Total	18.1	28.5	46.6	26.5	31.8	58.3

Supplementary Table 42. Comparison of cumulative net primary production (NPP, Pg C) among land cover types on areas covered by croplands during 2000 over different time periods under two climate change projections (RCP8.5, RCP4.5) when croplands are fertilized from 1950 to 2100 (Fertilized Crop)

Land Cover	RCP8.5			RCP4.5		
	2001-2050	2051-2100	2001-2100	2001-2050	2051-2100	2001-2100
Croplands	57.4	58.9	116.3	49.9	35.4	85.3
Pastures	0.3	1.0	1.3	0.1	0.3	0.4
Young Forests						
Boreal	0.5	0.7	1.2	6.4	12.5	18.9
Temperate	1.0	1.9	2.9	3.4	9.8	13.2
All	1.5	2.6	4.1	9.8	22.3	32.1
Old Forests						
Boreal	0.0	0.0	0.0	0.0	0.0	0.0
Temperate	0.0	0.0	0.0	0.0	0.0	0.0
All	0.0	0.0	0.0	0.0	0.0	0.0
Forests						
Boreal	0.5	0.7	1.2	6.4	12.5	18.9
Temperate	1.0	1.9	2.9	3.4	9.8	13.2
All	1.5	2.6	4.1	9.8	22.3	32.1
Temperate Grasslands	0.2	0.2	0.4	2.3	6.6	8.9
Temperate Shrublands	0.0	0.0	0.0	0.1	0.2	0.3
Tundra	0.0	0.0	0.0	0.0	0.2	0.2
Wetlands	0.0	0.1	0.1	0.5	1.2	1.7
Deserts	0.0	0.0	0.0	0.0	0.0	0.0
Total	59.4	62.8	122.2	62.7	66.2	128.9

Supplementary Table 43. Comparison of legacy N fertilizer effects on cumulative net primary production (NPP, Pg C) among land cover types on areas covered by croplands during 2000 over different time periods under the RCP 8.5 climate change projection for unfertilized croplands (Unfertilized Crops) and croplands fertilized from 1950 to 2000 (Pre-2001 Fertilized Crops)

Land Cover	Unfertilized Crops			Pre-2001 Fertilized Crops		
	2001-2050	2051-2100	2001-2100	2001-2050	2051-2100	2001-2100
Croplands	18.8	22.6	41.4	20.9	25.0	45.9
Pastures	0.3	0.8	1.1	0.4	0.8	1.2
Young Forests						
Boreal	0.5	0.7	1.2	0.5	0.7	1.2
Temperate	1.0	1.6	2.6	1.0	1.8	2.8
All	1.5	2.3	3.8	1.5	2.5	4.0
Old Forests						
Boreal	0.0	0.0	0.0	0.0	0.0	0.0
Temperate	0.0	0.0	0.0	0.0	0.0	0.0
All	0.0	0.0	0.0	0.0	0.0	0.0
Forests						
Boreal	0.5	0.7	1.2	0.5	0.7	1.2
Temperate	1.0	1.6	2.6	1.0	1.8	2.8
All	1.5	2.3	3.8	1.5	2.5	4.0
Temperate Grasslands	0.2	0.2	0.4	0.2	0.2	0.4
Temperate Shrublands	0.0	0.0	0.0	0.0	0.0	0.0
Tundra	0.0	0.0	0.0	0.0	0.0	0.0
Wetlands	0.0	0.1	0.1	0.0	0.1	0.1
Deserts	0.0	0.0	0.0	0.0	0.0	0.0
Total	20.8	26.0	46.8	23.0	28.6	51.6

Supplementary Table 44. Comparison of legacy N fertilizer effects on cumulative net primary production (NPP, Pg C) among land cover types on areas covered by croplands during 2000 over different time periods under the RCP 4.5 climate change projection for unfertilized croplands (Unfertilized Crops) and croplands fertilized from 1950 to 2000 (Pre-2001 Fertilized Crops)

Land Cover	Unfertilized Crops			Pre-2001 Fertilized Crops		
	2001-2050	2051-2100	2001-2100	2001-2050	2051-2100	2001-2100
Croplands	16.6	13.7	30.3	18.7	15.4	34.1
Pastures	0.1	0.3	0.4	0.1	0.3	0.4
Young Forests						
Boreal	6.7	11.3	18.0	6.7	12.0	18.7
Temperate	3.3	8.4	11.7	3.4	9.4	12.8
All	10.0	19.7	29.7	10.1	21.4	31.5
Old Forests						
Boreal	0.0	0.0	0.0	0.0	0.0	0.0
Temperate	0.0	0.0	0.0	0.0	0.0	0.0
All	0.0	0.0	0.0	0.0	0.0	0.0
Forests						
Boreal	6.7	11.3	18.0	6.7	12.0	18.7
Temperate	3.3	8.4	11.7	3.4	9.4	12.8
All	10.0	19.7	29.7	10.1	21.4	31.5
Temperate Grasslands	1.8	4.8	6.6	2.3	5.8	8.1
Temperate Shrublands	0.1	0.2	0.3	0.1	0.3	0.4
Tundra	0.1	0.1	0.2	0.0	0.2	0.2
Wetlands	0.6	1.1	1.7	0.6	1.1	1.7
Deserts	0.0	0.0	0.0	0.0	0.0	0.0
Total	29.3	39.9	69.2	31.9	44.5	76.4

Supplementary Table 45. Changes in coverage (million ha) among land covers/land uses for areas underlain by permafrost during the year 2000 over the 21st century under two climate change projections (RCP8.5, RCP4.5) and land-use transitions described by Hurtt et al.[11]

Land Cover	2000	RCP8.5		RCP4.5	
		2050	2100	2050	2100
Croplands	34	33	33	26	23
Pastures	117	115	116	95	96
Young Forests					
Boreal	25	57	95	43	56
Temperate	1	3	5	7	9
All	26	60	100	50	65
Old Forests					
Boreal	238	206	168	224	211
Temperate	9	7	5	5	3
All	247	213	173	229	214
All Forests					
Boreal	263	263	263	267	267
Temperate	10	10	10	12	12
All	273	273	273	279	279
Temperate Grasslands	33	36	35	55	57
Temperate Shrublands	2	2	2	4	4
Tundra	287	287	287	287	287
Wetlands	42	42	42	42	42
Deserts	127	127	127	127	127
Lakes	5	5	5	5	5
Glaciers	0	0	0	0	0
Total	920	920	920	920	920

Supplementary Table 46. Comparison of cumulative net ecosystem carbon balance (NECB, Pg C) among land cover types for areas underlain by permafrost during the year 2000 over different time periods under two climate change projections (RCP8.5, RCP4.5)

Land Cover	RCP8.5			RCP4.5		
	2001-2050	2051-2100	2001-2100	2001-2050	2051-2100	2001-2100
Croplands	-0.2	-0.3	-0.5	0.0	-0.1	-0.1
Pastures	-0.3	-0.2	-0.5	-0.1	-0.1	-0.2
Young Forests						
Boreal	-2.7	3.8	1.1	0.0	1.5	1.5
Temperate	0.1	0.2	0.3	0.3	0.6	0.9
All	-2.6	4.0	1.4	0.3	2.1	2.4
Old Forests						
Boreal	0.9	7.6	8.5	1.0	5.7	6.7
Temperate	0.2	0.2	0.4	0.1	0.1	0.2
All	1.1	7.8	8.9	1.1	5.8	6.9
Forests						
Boreal	-1.8	11.4	9.6	1.0	7.2	8.2
Temperate	0.3	0.4	0.7	0.4	0.7	1.1
All	-1.5	11.8	10.3	1.4	7.9	9.3
Temperate Grasslands	0.1	0.0	0.1	0.2	0.0	0.2
Temperate Shrublands	0.0	0.0	0.0	0.0	0.0	0.0
Tundra	0.9	-2.4	-1.5	0.8	0.2	1.0
Wetlands	0.1	0.4	0.5	0.2	0.3	0.5
Deserts	0.9	1.2	2.1	0.9	0.8	1.7
Total	0.0	10.5	10.5	3.4	9.0	12.4

Supplementary Table 47. Comparison of cumulative carbon sequestration in vegetation (VegC$_{NEW}$, Pg C) among land cover types for areas underlain by permafrost during the year 2000 over different time periods under two climate change projections (RCP8.5, RCP4.5)

Land Cover	RCP8.5			RCP4.5		
	2001-2050	2051-2100	2001-2100	2001-2050	2051-2100	2001-2100
Croplands	0.0	0.1	0.1	-0.1	0.1	0.0
Pastures	0.0	0.1	0.1	0.1	-0.1	0.0
Young Forests						
Boreal	-0.2	4.8	4.6	1.1	1.0	2.1
Temperate	0.3	-0.2	0.1	0.2	0.4	0.6
All	0.1	4.6	4.7	1.3	1.4	2.7
Old Forests						
Boreal	4.1	8.3	12.4	4.0	4.2	8.2
Temperate	0.2	0.3	0.5	0.3	-0.1	0.2
All	4.3	8.6	12.9	4.3	4.1	8.4
Forests						
Boreal	3.9	13.1	17.0	5.1	5.1	10.3
Temperate	0.5	0.1	0.6	0.5	0.3	0.8
All	4.4	13.2	17.6	5.6	5.5	11.1
Temperate Grasslands	0.1	0.0	0.1	0.1	0.1	0.2
Temperate Shrublands	0.0	0.0	0.0	0.1	-0.1	0.0
Tundra	0.8	0.7	1.5	0.5	0.3	0.8
Wetlands	0.1	0.7	0.8	0.4	0.1	0.5
Deserts	0.2	0.3	0.5	0.2	0.1	0.3
Total	5.6	15.1	20.7	6.9	6.0	12.9

Supplementary Table 48. Comparison of cumulative carbon sequestration in soil organic matter ($TotSOC_{NEW}$, Pg C) among land cover types for areas underlain by permafrost during the year 2000 over different time periods under two climate change projections (RCP8.5, RCP4.5)

Land Cover	RCP8.5			RCP4.5		
	2001-2050	2051-2100	2001-2100	2001-2050	2051-2100	2001-2100
Croplands	-0.2	-0.3	-0.5	0.2	-0.2	0.0
Pastures	-0.2	-0.3	-0.5	-0.1	-0.1	-0.2
Young Forests						
Boreal	-2.9	-1.0	-3.9	-1.2	0.5	-0.7
Temperate	-0.1	0.2	0.1	0.1	0.1	0.2
All	-3.0	-0.8	-3.8	-1.1	0.6	-0.5
Old Forests						
Boreal	-3.1	-0.9	-4.0	-3.0	1.4	.-1.6
Temperate	0.0	0.0	0.0	-0.3	0.4	0.1
All	-3.1	-0.9	-4.0	-3.3	1.8	-1.5
Forests						
Boreal	-6.0	-1.7	-7.9	-4.2	1.9	-2.3
Temperate	-0.1	0.2	0.1	-0.2	0.5	0.3
All	-6.1	-1.7	-7.8	-4.4	2.4	-2.0
Temperate Grasslands	0.0	0.0	0.0	0.0	0.0	0.0
Temperate Shrublands	0.0	0.1	0.1	0.0	0.0	0.0
Tundra	0.1	-3.1	-3.0	0.2	0.0	0.2
Wetlands	0.0	-0.5	-0.5	-0.1	0.1	0.0
Deserts	0.6	1.0	1.6	0.7	0.7	1.4
Total	-5.8	-4.8	-10.6	-3.5	2.9	-0.6

Supplementary Table 49. Comparison of cumulative net nitrogen mineralization (NetNMin, Tg N) among land cover types for areas underlain by permafrost during the year 2000 over different time periods under two climate change projections (RCP8.5, RCP4.5)

Land Cover	RCP8.5			RCP4.5		
	2001-2050	2051-2100	2001-2100	2001-2050	2051-2100	2001-2100
Croplands	41.2	86.6	127.8	28.3	53.8	82.1
Pastures	136.1	161.0	297.1	128.1	131.4	259.5
Young Forests						
Boreal	102.3	264.8	367.1	107.3	157.0	264.3
Temperate	7.0	14.5	21.5	16.9	30.7	47.6
All	109.3	279.3	388.6	124.2	187.7	311.9
Old Forests						
Boreal	237.9	294.6	532.5	257.8	293.7	551.5
Temperate	23.3	22.9	46.2	20.8	12.0	32.8
All	261.2	317.5	578.7	278.6	305.7	584.3
Forests						
Boreal	340.2	559.4	899.6	365.1	450.7	815.8
Temperate	30.3	37.4	67.7	37.7	42.7	80.4
All	370.5	596.8	967.3	402.8	493.4	896.2
Temperate Grasslands	44.7	51.5	96.2	58.8	86.5	145.3
Temperate Shrublands	2.1	1.9	4.0	3.7	4.7	8.4
Tundra	73.7	87.6	161.3	72.2	85.1	157.3
Wetlands	24.2	38.3	62.5	25.9	28.4	54.3
Deserts	9.8	8.6	18.4	11.2	9.0	20.2
Total	702.3	1,032.3	1,734.6	731.0	892.3	1,623.3

Supplementary Table 50. Biome-specific parameters used to simulate net N mineralization[a] and biological N fixation[b] in Northern Eurasian ecosystems

Biome	*N*immcut	*N*imm1a	*N*imm1b	*N*imm2a	*N*imm2b	*ASym:BNfix*
Polar Desert	0.557	-40.888	31.397	-24.919	22.507	1.00
Tundra	0.551	-30.092	22.195	-13.973	13.310	1.00
Boreal Needle-leaf Evergreen Forest	0.442	-77.275	39.346	-9.621	9.413	1.00
Boreal Needle-leaf Deciduous Forest	0.511	-12.616	7.696	-2.127	2.340	1.00
Boreal Broadleaved Deciduous Forest	0.465	-29.165	15.242	-3.727	3.417	1.00
Shrubland	0.589	-46.293	42.508	-60.685	50.989	0.35
Grassland	0.471	-13.207	15.981	-22.463	20.339	0.90
Temperate Needle-leaf Evergreen Forest	0.549	-9.116	6.349	-3.334	3.173	0.50
Temperate Broadleaved Deciduous Forest	0.485	-10.782	6.392	-2.506	2.382	0.50
Xeromorphic Woodland and Shrublands	0.365	-4.088	4.769	-5.947	5.448	0.35
Boreal Wooded Wetland	0.442	-77.275	39.346	-9.621	9.413	1.00
Boreal Non-Wooded Wetland	0.551	-30.092	22.195	-13.973	13.310	1.00
Temperate Wooded Wetland	0.485	-10.782	6.392	-2.506	2.382	0.50
Temperate Non-Wooded Wetland	0.471	-13.207	15.981	-22.463	20.339	0.90
Coastal Salt Marsh	0.471	-13.207	15.981	-22.463	20.339	0.90
Inland Salt Marsh	0.471	-13.207	15.981	-22.463	20.339	0.90
Crops	0.471	-13.207	15.981	-22.463	20.339	0.90
Pasture	0.471	-13.207	15.981	-22.463	20.339	0.90

[a]The parameter *N*imm is estimated as a linear function of soil texture [*N*imm = *N*imma(psiplusc) + *N*immb] where psiplusc is the sum of the proportion silt plus the proportion clay in the soil. From calibrations, it has been found that one linear relationship holds up to a threshold point (*N*immcu*t*), but a different linear relationship holds above the threshold. Thus, the parameters *N*imm1a and *N*imm1b describe the linear relationship for soil textures below *N*immcut, and parameter *N*imm2a and *N*imm2b describe the linear relationship for soil textures above the *N*immcut.

[b]Biological N fixation is divided into asymbiotic N fixation or symbiotic N fixation based on the parameter *ASym:BNfix* that describes the proportion of biological N fixation contributed by asymbiotic N fixation.

Supplementary References

1. FAO. *Global Forest Resources Assessment 2015: Desk reference.* (Food and Agriculture Organization of the United Nations, 2015)

2. Goodale, C.L. et al. Forest carbon sinks in the Northern Hemisphere. *Ecol. Appl.* **12,** 891–899 (2002).

3. Pan, Y. et al. A large and persistent carbon sink in the world's forest. *Science* **333,** 988-993 (2011).

4. Thurner, M. et al. Carbon stock and density of northern boreal and temperate forests. *Global Ecology and Biogeography* **23,** 297–310 (2014).

5. Tarnocai, C. et al. Soil organic carbon pools in the northern circumpolar permafrost region. *Glob. Biogeochem. Cycles* **23,** GB2023 (2009).

6. Stolbovoi, V. Carbon pools in tundra soils of Russia: improving data reliability. In *Global Climate Change and Cold Regions Ecosystems* (eds. Lal, R., J. M. Kimble, J. M., & B. A. Stewart, B. A.) 39-58 (Lewis, 2000)

7. Dolman, A. J. et al. An estimate of the terrestrial carbon budget of Russia using inventory-based, eddy covariance and inversion methods. *Biogeosciences* **9,** 5323-5340 (2012).

8. Hayes, D. J. et al. Is the northern high-latitude land-based CO_2 sink weakening? *Glob. Biogeochem. Cycles* **25,** GB3018 (2011).

9. Schaphoff, S., Reyer, C. P. O., Schepaschenko, D., Gerten, D. & Shvidenko A. Tamm review: observed and projected climate change impacts on Russia's forests and its carbon balance. *Forest Ecol. Manag.* **361,** 432-444 (2016).

10. Saeki, T. et al. Carbon flux estimation for Siberia by inverse modeling constrained by aircraft and tower CO_2 measurements. *J. Geophys. Res.* **118,** 1100-1122 (2013).

11. Hurtt, G. C. et al. Harmonization of land-use scenarios for the period 1500–2100: 600 years of global gridded annual land-use transitions, wood harvest, and resulting secondary lands. *Climatic Change* **109,** 117-161 (2011).
